# Humans and seasonal climate variability threaten large-bodied coral reef fish with small ranges

**DOI:** 10.1038/ncomms10491

**Published:** 2016-02-03

**Authors:** C. Mellin, D. Mouillot, M. Kulbicki, T. R. McClanahan, L. Vigliola, C. J. A. Bradshaw, R. E. Brainard, P. Chabanet, G. J. Edgar, D. A. Fordham, A. M. Friedlander, V. Parravicini, A. M. M. Sequeira, R. D. Stuart-Smith, L. Wantiez, M. J. Caley

**Affiliations:** 1 Australian Institute of Marine Science, PMB No. 3, Townsville MC, Townsville, Queensland 4810, Australia; 2 The Environment Institute and School of Biological Sciences, The University of Adelaide, Adelaide, South Australia 5005, Australia; 3 UMR 9190 MARBEC, IRD-CNRS-IFREMER-UM, Université de Montpellier, 34095 Montpellier, France; 4 Australian Research Council Centre of Excellence for Coral Reef Studies, James Cook University, Townsville, Queensland 4811, Australia; 5 Institut de Recherche pour le Développement, UMR ‘Entropie', LABEX Corail, Université de Perpignan, 66000 Perpignan, France; 6 Marine Programs, Wildlife Conservation Society, Bronx, New York 10460, USA; 7 Institut de Recherche pour le Développement, UMR ‘Entropie', LABEX Corail, BP A5–98848 Nouméa, New Caledonia, France; 8 NOAA, National Marine Fisheries Service, Pacific Islands Fisheries Science Center, 1125B Ala Moana Blvd, Honolulu, Hawaii 96814, USA; 9 Institut de Recherche pour le Développement, UMR ‘Entropie', LABEX Corail, BP 50172, CS 41095, FR-97495 Ste Clotilde, Reunion Island, France; 10 Institute for Marine and Antarctic Studies, University of Tasmania, Private Bag 49, Hobart, Tasmania 7001, Australia; 11 Pristine Seas, National Geographic Society, Washington DC 20036, USA; 12 Fisheries Ecology Research Lab, University of Hawaii at Manoa, Honolulu, Hawaii 096822, USA; 13 Centre de Synthèse et d'Analyse sur la Biodiversité (Fondation pour la Recherche en Biodiversité), Immeuble Henri Poincaré, Domaine du Petit Arbois, Aix-en-Provence 13100, France; 14 CRIOBE, USR 3278 CNRS-EPHE-UPVD, LABEX Corail, University of Perpignan, 66860 Perpignan, France; 15 IOMRC and The UWA Oceans Institute, School of Animal Biology and Centre for Marine Futures, The University of Western Australia M470, 35 Stirling Highway, Crawley, Western Australia 6009, Australia; 16 Research Unit LIVE (EA4243), University of New Caledonia, Noumea, New Caledonia 98851, France

## Abstract

Coral reefs are among the most species-rich and threatened ecosystems on Earth, yet the extent to which human stressors determine species occurrences, compared with biogeography or environmental conditions, remains largely unknown. With ever-increasing human-mediated disturbances on these ecosystems, an important question is not only how many species can inhabit local communities, but also which biological traits determine species that can persist (or not) above particular disturbance thresholds. Here we show that human pressure and seasonal climate variability are disproportionately and negatively associated with the occurrence of large-bodied and geographically small-ranging fishes within local coral reef communities. These species are 67% less likely to occur where human impact and temperature seasonality exceed critical thresholds, such as in the marine biodiversity hotspot: the Coral Triangle. Our results identify the most sensitive species and critical thresholds of human and climatic stressors, providing opportunity for targeted conservation intervention to prevent local extinctions.

## Abstract (web summary)

Knowing which species traits may confer resilience to human-mediated stressors will help predict future impacts on biodiversity. Here, Mellin *et al*. show that large bodied fish with small geographic ranges are disproportionately affected by the negative impacts of human disturbance and climate variability.

## Graphical abstract

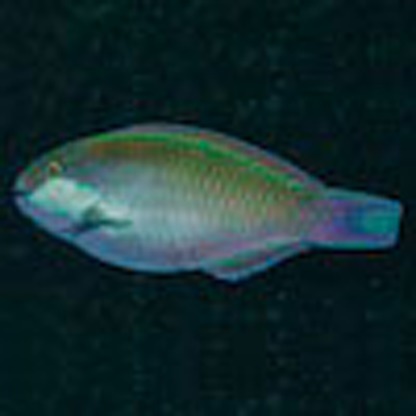

Understanding the processes that generate and maintain species occurrence is essential for designing interventions to mitigate biodiversity loss. Yet, identifying the drivers of species occurrence patterns is challenging, partly due to confounding natural and human-mediated effects^1^. The peak in marine biodiversity observed in the Coral Triangle has been explained by several non-mutually exclusive hypotheses that involve the roles of energy^2^, habitat area^3^, biogeography^4^ and geometric constraints on species range sizes^5^,^6^. By contrast, the impact of cumulative human pressure (combining fisheries, human density, urban development and climate change metrics^7^,^8^,^9^) on global marine biodiversity patterns has long been overlooked. With the recent development of multifaceted metrics of cumulative anthropogenic pressures^7^,^8^,^9^, it is now possible to disentangle their impacts from ecological and evolutionary determinants of biodiversity patterns. Doing so now is critical to help identify and prioritize tractable options for conservation actions to mitigate accelerating human impacts on biological communities.

Most studies investigating biodiversity patterns implicitly consider species as comparable units^10^. However, the ecological roles of species also matter, with many species—particularly those with restricted geographical ranges^11^ —supporting unique and indispensable functions^12^. Furthermore, different subsets of species respond differently to environmental and human stressors^13^, most often in non-linear ways with critical thresholds. For example, fishing and climate change can differentially impact the abundance and biomass of fishes depending on their body sizes^14^,^15^. Human pressure also has the potential to reduce species abundances, which in turn can cause ecological extinction (that is, when large population declines prevent species from performing their ecological roles)^16^, local extinction (that is, extirpation)^17^ and, ultimately, global extinction^18^. Yet compared with human-mediated decreases in abundances, the loss of species occurrences under human pressure remains largely unknown for coral reef fishes over broad spatial scales^19^, and cannot be inferred from reduced local abundance because abundance and occupancy are unrelated for fishes on coral reefs^20^. Thus, the extent to which human stressors shape species occurrence patterns within their geographical range, once natural and biogeographic factors have been accounted for, requires urgent assessment—as does the extent to which these relationships might be modulated by biological traits such as body size.

Our analyses of coral reef fishes combined data from 906 locations across the Indo-Pacific along with biological traits^21^,^22^, including maximum adult total length, trophic group, home range size, mobility, diel activity, schooling behaviour and geographical range size^6^ estimated as the extent of occurrence^23^. Coral reef fishes are ideal for examining correlates of broad-scale occurrence patterns because they (i) are species-rich (>4,800 species within the Indo-Pacific^24^), (ii) respond to environmental gradients at multiple scales^6^, particularly in comparison to most other vertebrate taxa and (iii) include a wide range of body sizes (from a few cm to>3 m total length), life histories, and reproductive strategies. We focused our analyses on 241 well-known and easily detected species of coral reef fishes that were consistently sampled on reefs across the Indo-Pacific and that encompass a wide spectrum of biological traits and geographical range sizes. We assessed how occurrence patterns in coral reef fishes respond to multiple indices of human pressure, which included past and present threats^7^, the human impact index^8^ mostly reflecting intense artisanal fishing and dense human populations and the ocean health index^9^. Using machine-learning techniques^25^ combined with detectability and null permutation models, we identified the main correlates of occurrence for each species and their associated thresholds among (i) indices of human pressure, (ii) energy proxies, including sea surface temperature and primary productivity, (iii) habitat area (both present and historical^26^) and (iv) biogeography, including distances to land masses and the Coral Triangle. We considered energy proxies to be potentially important because temperature influences species occurrence through phenological and physiological contraints^27^, while higher primary productivity supports larger populations that more effectively resist extinction^2^. Reef area increases the probability of colonization from neighbouring reefs, while biogeographic isolation from the main coral reef habitats accounts for large-scale connectivity and long-term persistence through dispersal^4^,^28^.

Using the most extensive data set on tropical reef fish occurrences (presences and absences) across the entire Indo-Pacific, we tested whether the vulnerability of fish occurrence to human pressure is modulated by fish body size and geographical range size, while controlling for energy, area and biogeography. We show that (i) the occurrence of relatively large-bodied tropical reef fishes (>50 cm total length) is strongly and negatively associated with cumulative human pressure and, to a lesser extent, negatively associated with temperature seasonality; and that (ii) this effect is most pronounced for the large-bodied species with the relatively smallest geographical ranges (that is, within the first quartile of geographic range sizes; *n*=13).

## Results and Discussion

### Quality control of the fish data

We found no evidence of any consistent data source or temporal effects in the fish occurrence data (permanova; 999 permutations, *P*>0.05). Conversely, we found evidence for an effect of body size and behaviour on fish detectability (Supplementary Table 1; *model*1; weight of Akaike's information criterion corrected for sample samples (*w*AIC_*c*_)>0.9). Detectability decreased with maximum body size (mean effect size±s.e.=−0.006±0.001), high mobility (−0.289±0.053), a solitary behaviour (−0.562±0.049) and high level in the water column (−1.035±0.135). However, the models including geographic variation (*model*2) or important correlates (*model*3) received little support (*w*AIC_*c*_<0.1; Supplementary Table 1), and residuals of the first model were evenly distributed within the study area (Supplementary Fig. 1A). These results suggest that even though detectability differed among species, this effect was evenly distributed among samples and within the correlate space, and did not affect the relationships between different correlates and fish occurrence patterns. We also found no effect of fishing intensity on the probability of recording false absences, either for all species or targeted/large ones (Supplementary Fig. 1B). Locations with missing fish data were evenly distributed across the correlate space as indicated by a principal component analysis (Supplementary Fig. 2) based on correlates related to biogeography, energy, area and human pressure, suggesting that species–correlate relationships inferred by the models were not influenced by missing data.

### Main correlates of fish occurrence patterns

**Figure 1:**
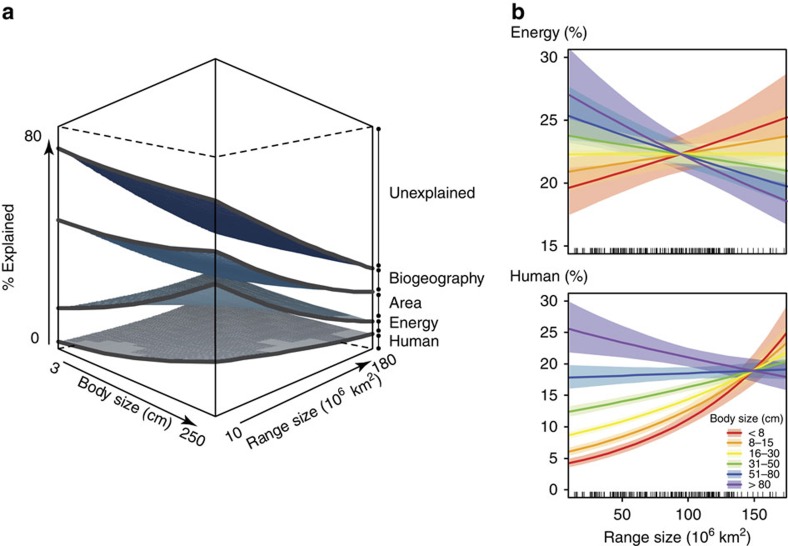
Influence of body size, geographic range size and their interactions, on fish occurrence patterns. (**a**) Percent variation explained in species probability of occurrence by biogeography, energy (temperature and primary productivity), area, and human pressure, and as a function of maximum adult total length (body size, in cm; log scale) and geographic extent of occurrence (geographic range size, in 10^6^ km^2^). (**b**) Relationship between geographic range size and the relative contribution of energy (top) and human pressure (bottom) to the variation explained in fish occurrence patterns as a function of maximum adult body size. Envelopes indicate 95% confidence intervals.

**Figure 2:**
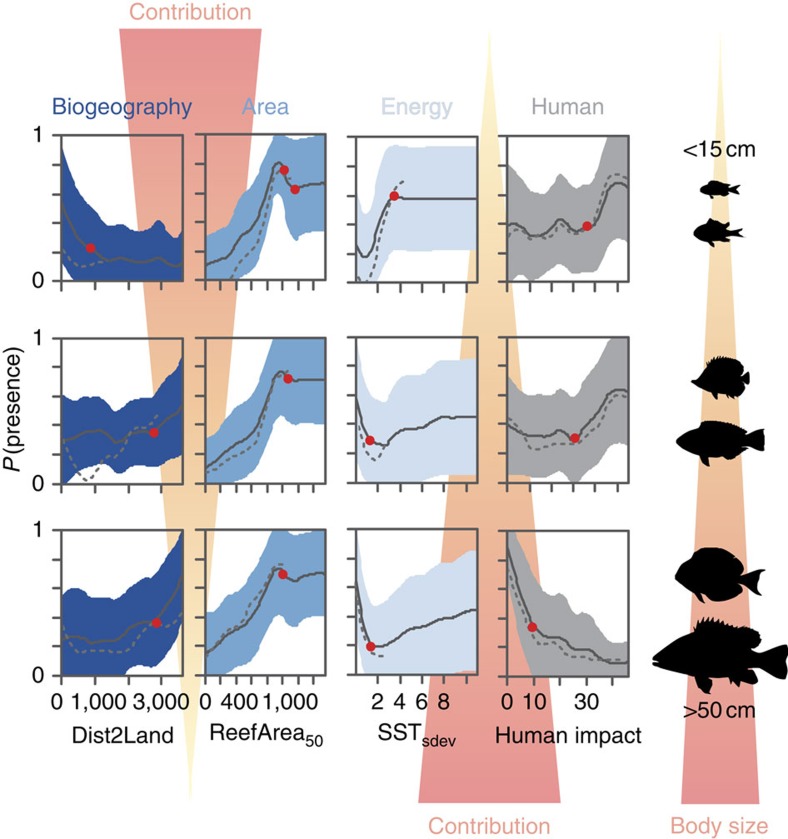
Predicted probability of occurrence and associated thresholds for species of increasing body size in response to biogeography, energy, area-related correlates and human impact. Only the relationships with the strongest correlates in each category are shown, with *Dist2Land*: distance to nearest land mass, in km; *ReefArea*_*50*_: reef area, in km^2^; SST_sdev_: seasonal deviation (that is, seasonality) in sea surface temperature, in °C. For each plot, the continuous line represents the mean effect across species and the envelope indicates the 95% confidence interval. Red dots indicate critical thresholds in the mean effect across species (Davies test, *P*<0.05). Dotted lines show the response of small-ranging species (first quartile of geographic range sizes), truncated to represent only the range of values where they occur (up to the 98th percentile). Contribution daggers reflect the change in correlate contribution as body size increases.

Human pressure and energy had disproportionately large effects on the occurrence of large-bodied species (>50 cm; Fig. 1a), with negative relationships between occurrence probability and both human impact and temperature seasonality (the most important human pressure and energy correlates; Fig. 2). By contrast, occurrences of smaller-bodied species were best explained by biogeographical correlates (Fig. 1a), with a positive relationship between reef area and occurrence probability (Fig. 2). Among large-bodied species, which tend to have large geographic ranges (Supplementary Fig. 2), those with relatively smaller ranges (<90 × 10^6^ km^2^; with a mean range of 57 × 10^6^ km^2^, equivalent to half that of an average large-bodied fish) were particularly and negatively affected by human pressure and energy (Fig. 1a, foreground edge of the cube and Fig. 2, dotted lines). The total amount of variation explained in occurrence patterns declined from 71% for the smallest species to 39% for the largest, with 5 and 10%, respectively associated with human pressure (Supplementary Fig. 3) (that is, 8–23% in terms of relative contribution; Supplementary Fig. 4). Of the total variation explained in occurrence patterns among species (Supplementary Fig. 5), body size combined with range size explained 46% (Supplementary Table 2). These patterns were consistent with the typical trend of decreasing probability of occurrence (concomitant with decreasing abundance^29^) as body size increases^30^,^31^ (Supplementary Fig. 6). Other biological traits (for example, diet, home range size) did not explain additional variation in occurrence patterns (Supplementary Fig. 7; Supplementary Table 3).

These patterns differed from those expected under a null model of randomized occurrences within each species' geographical range. Null boosted regression trees converged for 121 species only (∼ 50% of all species considered) and explained between 1.0 and 23.1% deviance in fish occurrence patterns (mean±standard deviation=5.3±4.7%). We found no evidence for a relationship between the total deviance explained and body size (Supplementary Fig. 8; Supplementary Table 4), or between the relative contributions of the different correlates and body or range sizes (Supplementary Fig. 9; Supplementary Table 4).

Our findings indicate an increasing negative influence of human pressure and temperature seasonality on fish occurrence as body size increases and species range size decreases. Small species tend to disperse less than large ones^32^, and their occurrences are primarily a function of biogeography, suggesting that isolation from source populations (decreasing dispersal rates) plays an important role in shaping their regional-scale occurrences^28^. For larger species with slower growth rates, fishing or habitat degradation can more effectively reduce fish stocks, affecting local and regional patterns of population size and biomass^15^. Our results show that human pressure and temperature seasonality can potentially affect not only local population size, but also regional occurrence patterns of large-bodied and small-ranging fishes in particular.

### Human impact and fish occurrence patterns

**Figure 3:**
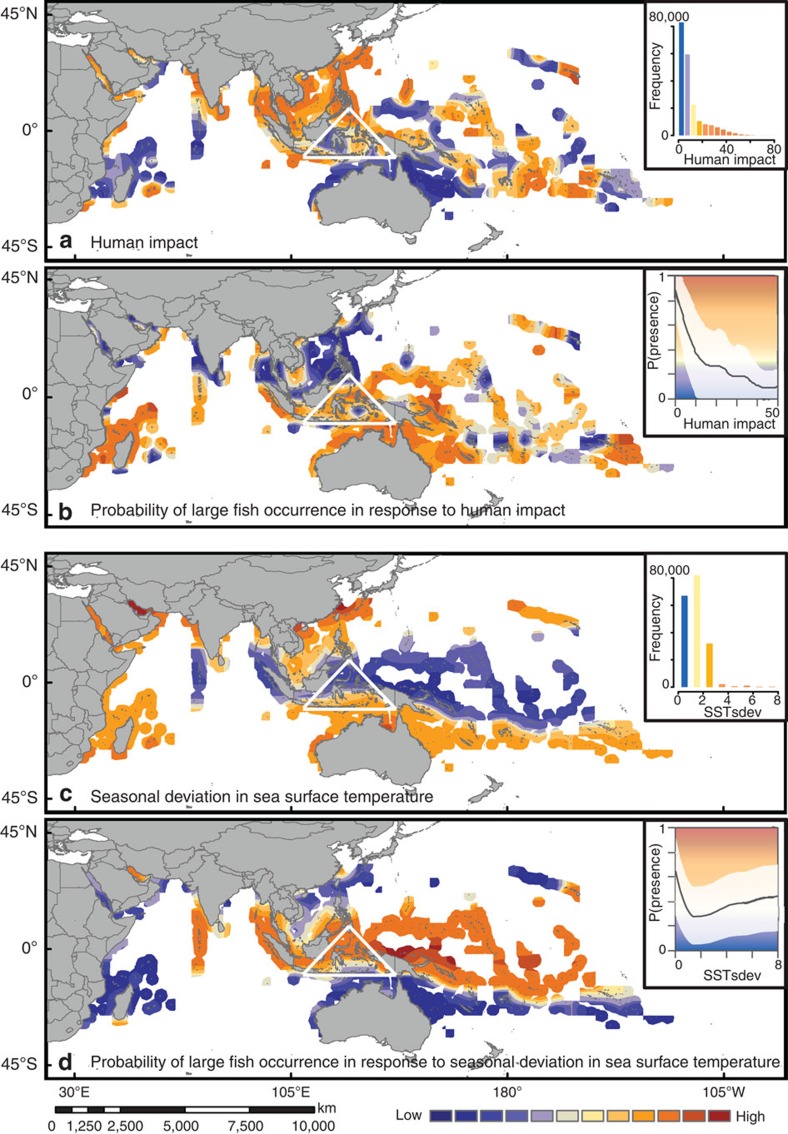
Maps of human and climate seasonal variability, and predicted probabilities of large fish occurrence. (**a**) Human impact and (**b**) predicted probability of occurrence of large fishes (body size >50 cm) within Indo-Pacific reefs in response to human impact; (**c**) Seasonal deviation (that is, seasonality) in sea surface temperature (SST_sdev_) and (**d**) predicted probability of occurrence of large fishes in response to SST_sdev_. (**a**,**c**) Insets show the distribution of (**a**) human impact and (**c**) SST_sdev_ across the study area. (**b**,**d**) Insets show the partial effect of (**b**) human impact and (**d**) SST_sdev_ averaged across large fishes and the 95% confidence interval. The triangle indicates the location of the Coral Triangle. On each plot, the mid-point of the colour scale corresponds to the critical threshold in the mean effect across species (Davies test, *P*<0.05).

Large fishes tend to occur less frequently on human-impacted reefs (Fig. 3b), highlighting a gradient of increasing occurrence with distance from the Coral Triangle. This pattern contrasts with the well-known gradient in marine biodiversity^2^ that peaks in the Coral Triangle. Large species occurrence was negatively related to the human impact index^8^ (the most important human pressure variable we examined). This pattern was stronger for large-bodied, relatively small-ranging fishes (Fig. 2) for which the contribution of human impact was greatest (Fig. 1a). Owing to the non-linear and negative relationship between human impact and the occurrence of large-bodied fishes, high occurrence probabilities of large-bodied and small-ranging species were only observed where human impact was low to moderate (Fig. 2 and Supplementary Fig. 10) with critical thresholds (Table 1). This means that under such thresholds, even a small reduction in human impact was associated with a much higher probability of encountering those large fish species. More specifically, reefs subject to a human impact index >9.9 (equivalent to conditions encountered in the Solomon Archipelago and currently representing 30% of all Indo-Pacific coral reefs) have a probability <0.3 of hosting large fishes. This low probability of occurrence represents a 60% reduction (67% for large-bodied, small-ranging fishes) from the greatest occurrence probabilities (0.7) that characterize less impacted reefs in New Caledonia or on the Great Barrier Reef (Fig. 3). This spatial gradient of large fish occurrence probabilities due to decreasing human impact from the Coral Triangle towards the south-west Pacific contrasts with the gradient of fish species richness^3^ and corroborates recent results^33^ showing that large species, and large-bodied and small-ranging fishes in particular, might contribute only marginally to high local species richness within the Coral Triangle, but much more to the richness of less-diverse assemblages at its periphery. Conversely, human pressure was in general positively, but weakly, associated with the occurrence of small-bodied species (Fig. 2), with small fishes tending to be more frequent on impacted reefs (that is, subject to a human impact >36.4; Table 1). This result corroborates previous studies documenting an increase in the relative abundances of small fishes on highly disturbed or fished reefs^34^,^35^,^36^.

### Climate seasonal variability and fish occurrence patterns

The probability of occurrence of large (and small-ranging) fishes was also greater where sea surface temperatures were less seasonally variable (Fig. 2). This generally resulted in higher probabilities of occurrence at low latitudes (Fig. 3d); although large species, which tend to have larger ranges than smaller ones^32^, are still likely to occur in more variable environments than smaller species (as a consequence of their generally larger ranges)^37^. Temperature can affect marine organisms through (i) an advanced onset of growing or breeding season due to earlier springs and later autumns, (ii) a temporal mismatch between food requirement and availability and (iii) temperature extremes exceeding thermal tolerance thresholds^38^,^39^,^40^. Our results support the contention that large-bodied species are not only more susceptible to over-fishing^14^, they are also more sensitive to climate-induced shifts in the timing of seasonal events, which are exacerbated in habitats of low temperature seasonality such as the Coral Triangle^40^. Even for tropical species, temperature seasonality is a major phenological driver (for example, spring temperatures trigger spawning aggregations in the coral trout *Plectropomus leopardus*^41^). Any future shifts in such seasonal events could thus have pronounced deleterious effects on recruitment, in particular if environmental conditions are then unsuitable for larval survival and growth^38^. Direct effects of climate change such as seasonal shifts and climate velocity are particularly affecting the Coral Triangle^40^, leading to forecasts of high extirpation rates, redistribution of biodiversity, and the formation of no-analogue communities in the near future^42^,^43^. While we found limited evidence for temporal variation in temperature over the 12 years considered here (Southern Oceania only; Supplementary Fig. 11), recent evidence suggests that, in addition to seasonal shifts, the frequency of both El Niño and La Niña events is increasing, resulting in more frequent temperature extremes^44^,^45^. Finally, future studies should examine other critical aspects of climate change not included here such as ocean acidification^46^ and tropical cyclones^47^,^48^, and their potential impact on coral reef fishes (now and in the future), which would require data at finer spatial and temporal resolutions than those used in this study.

### Vulnerability of large-bodied fishes with small ranges

Large-bodied, small-ranging fishes represent only 7% of all the species we examined here (Supplementary Data 1), yet because of their unique functional roles and ecosystem services they provide^11^, their greater sensitivity to human pressure could have cascading effects on entire reef ecosystems. Some of these species are commercially exploited and sustain local artisanal fisheries in many developing nations, but their conservation status remains largely unassessed^49^. This oversight is partly due to the recent focus of conservation strategies to protect particular functional groups like herbivores, which are deemed to play an essential role in the prevention of phase shifts from coral- to algae-dominated states^50^,^51^. However, we found no evidence that herbivore occurrences were particularly affected by human pressure at the scale of the entire Indo-Pacific, possibly because the broad spatio-temporal scales at which these data were aggregated masked the importance of recent environmental changes at individual reefs, to which trophic affiliation often regulates species responses^50^,^51^. Instead, the combination of large body size (usually associated with slow growth rates) and restricted geographical range (suggesting limited physiological tolerance) puts these species at higher risk of local extinction, irrespective of their other traits.

Large-bodied, small-ranging fishes are likely to be particularly susceptible to local extinction over the coming decades because (i) their restricted geographical ranges imply that any additional stressors would have a disproportionate effect on their occurrence patterns compared with more widely distributed species, and (ii) such stressors are expected to increase over the coming decades. The vulnerability of coral reef fishes to global change might thus depend strongly on the interplay between the body sizes and geographic range sizes of these species. Our results strongly indicate that these potential drivers of extinction urgently need to be incorporated into conservation strategies aimed at minimizing local biodiversity loss and thus maximizing ecosystem resilience to future disturbances.

## Methods

### Fish occurrence data and biological traits

We obtained fish occurrence (presence/absence) data from 9,828 samples, of which 93% were transects and the remaining point counts, from 906 locations of similar spatial extent within the Indo-Pacific (Supplementary Fig. 12; Supplementary Table 5). These data were collected by underwater visual census based on either fixed-length belt transects^52^ or stationary point counts^53^ in shallow reef habitats (depth 0–30 m), where all fishes sighted in the survey area were recorded on an underwater slate by divers. A detailed description of the methods used for fish sampling is provided in Supplementary Table 5 and references therein.

We selected 241 species from 10 families (Supplementary Data 1) for analysis based on the following criteria: (i) they satisfied minimum detection criteria (that is, we excluded cryptic and rare species, and those <3 cm total maximum length), and (ii) they covered the broadest range of life-history traits^21^ and geographical ranges^32^ possible with minimal uncertainty around those estimates. We used an independent data set of expert-verified checklists^24^ to delineate each species' geographical range (convex hull, defined as the smallest convex polygon containing all species records) as the basis for calculations of geographic range sizes (i.e., extent of occurrence^23^; in 10^6^ km^2^). For each species, we calculated range size (defined as the total area of the convex hull minus total land area) in ArcGis 10.0 using a global equal-area Behrmann projection. For each species, we also collated the following life-history traits^21^,^22^: trophic group, body size (i.e., maximum adult total length, in cm), home range, mobility, diel activity pattern and schooling behaviour. Some species were occasionally not sampled because two data sets (WCS and PROCFish; Supplementary Table 5) used a restricted species list. This resulted in 8% of all records missing; therefore, we did not use these records during model calibration and verified that missing data did not affect our analyses (see ‘Missing fish data' section).

### Environmental and anthropogenic variables

We selected environmental correlates related to major hypotheses attempting to explain variation in fish diversity in previous studies and based on general ecological theory. Considering the spatially aggregated nature of our data, we focused on large-scale environmental correlates that were mostly relevant to our analysis of occurrence patterns across locations (typically 10–100 km apart), instead of finer-scale environmental correlates (for example, benthic cover) that were inconsistently available across all sites. We considered the following large-scale environmental correlates: (i) biogeography, because it is related to dispersal rates through local connectivity; (ii) habitat area, because it is related to the probability of colonization from neighbouring reefs or patches within reefs and export to other reefs; and (iii) energy, because its availability can constrain species occurrence based on their physiological tolerances and because greater energy availability can sustain larger populations. We also considered a range of proxies for (iv) human pressure (past and present threat^7^, human impact^8^ and ocean health index^9^) to account for potential pressures on coral reefs resulting from fisheries exploitation, pollution, urban development, aquaculture and past thermal stress (Supplementary Table 6), which can affect coral reef ecosystems at a global scale^2^,^54^. We extracted these environmental data from global data sets (Supplementary Table 6) and matched them to the locations where fishes were sampled. Details for individual correlates and data sources are provided below.

(i) We used biogeographic correlates as proxies for connectivity and relative position within a species' geographical range, and included the shortest distance (km) to the nearest landmass^54^ >10^5^ km^2^, the shortest distances to the edge of the continental shelf (km) and to the nearest species range margin (km). Following previous studies^6^, we defined the continental shelf as the sea bottom to 200 m depth using the Shuttle Radar Topography Mission SRTM30_PLUS bathymetry (http://topex.ucsd.edu/WWW_html/srtm30_plus.html). We also included the relative distance to the nearest range margin, defined as the absolute distance to the range margin (km) divided by half the distance between the farthest two range endpoints^32^; the relative distance is therefore 0 at the range margin, and 1 halfway between the farthest range endpoints. To account for the relative position with the range, we calculated the relative longitude and latitude as varying between −1 for the easternmost (or southernmost) endpoint and 1 for the westernmost (or northernmost) endpoint. Finally, we included the distance to the Indo-Australian Archipelago (km) where fish diversity peaks^5^.

(ii) Area correlates included the area of the sampled reef (km^2^) and its perimeter (km; to account for increased habitat availability on reefs with complex shapes), total reef area within 10- and 50-km kernels centred on the sampling location (to account for potential diversity of nearby reefs and its possible influence on the sampled reef through local connectivity), and the total area of continental shelf within a 50-km kernel. We considered shelf area to be an appropriate estimate of historical habitat availability because it provides an approximate estimate of the coastal waters during the Pleistocene low sea-level stands^5^. We chose 10 and 50 km as cut-off kernel radii^28^ because such distances (i) are representative of larval dispersal distances for a variety of reef fishes, typically estimated as ranging between of 0 and 100 km and (ii) resulted in the most complete landscape description within the vicinity of reefs while minimizing pseudo-replication due to kernel overlap among neighbouring locations. We calculated area correlates in ArcGIS 10.0 from a reef contour shapefile^7^ derived from remote sensing.

(iii) We used energy correlates to account for both kinetic (temperature) and potential energy (primary productivity); these included sea surface temperature (SST, in °C) and chlorophyll *a* concentration (Chl *a*, in mg m^−3^). At a global scale, SST and Chl *a* are strongly correlated with other satellite-derived energy proxies^55^ such as photosynthetically active radiation and light attenuation, and have commonly been used as predictors of fish diversity^2^,^54^ because they facilitate larger population sizes (for example, through enhanced larval survival) thereby reducing the probability of local extinctions and supporting the persistence of niche specialists^2^. For both SST and Chl *a*, we calculated the climatological (annual) mean, winter and summer means and the annual thermic variation (seasonality), defined as the s.d. in monthly means^56^ and averaged across years, at sampled locations from MODIS Aqua monthly climatology (between 2002 and 2013) at a 9-km resolution. Seasonality describes within year variation (in SST or Chl *a*), or how different seasonal conditions are throughout the year.

(iv) Human pressure correlates included past and present threat^7^, human impact^8^, ocean health index^9^ to account for potential pressures on coral reefs resulting from fisheries exploitation, pollution, urban development, aquaculture and climatic stress (Supplementary Table 6), which can affect coral reef ecosystems at a global scale^2^,^54^. Many coastal centres of high species richness overlap with regions of medium to high human impact^2^. Human population density correlates with fishing and coastal development; and land-use stressors disproportionately impact fish biomass at more diverse reefs^54^. Specifically, the present local threat to coral reefs^7^ (from ‘low' to ‘very high') combines threats from overfishing and destructive fishing, coastal development, watershed- and marine-based pollution and damage. The present integrated threat that accounts for past climatic stress^7^ (from ‘low' to ‘very high') additionally incorporates severe thermal stress potentially responsible for mass coral bleaching events between 1997 and 2008.

For coral reefs, the human impact^8^ model is mostly driven by three main factors: artisanal fishing (FAO-based artisanal catch rates), climate change (frequency and intensity of sea temperature anomalies between 1985 and 2005) and direct human impact (population density). The human impact model also incorporates other factors, such as commercial fishing, pollution and species invasions, although these are relatively less important for coral reefs compared with other marine ecosystems^8^. The human impact model has been validated for coral reefs with cumulative human impact being highly correlated with the current condition of coral reefs worldwide and based on the relative abundance of a suite of indicator species (see Online Material^8^).

The ocean health index^9^, available for every coastal country, reflects ten diverse public goals for a healthy coupled human-ocean system. These goals include (i) food provision, including fisheries and mariculture, (ii) artisanal fishing opportunity, (iii) natural products, (iv) carbon storage, (v) coastal protection, (vi) tourism and recreation, (vii) coastal livelihoods and economies, (viii) sense of place (including iconic species and lasting special places), (ix) clean waters and (x) biodiversity, including habitats and species. The main ocean index score is a synthetic metric that results from the aggregation of these public goals. Each of these ten goals (and their sub-components) comprising the index can be considered separately or aggregated into the overall score. The overall index score for the global ocean is 60 out of 100, with non-random spatial variation^9^. Because conclusions based on a single goal will deviate from those derived from the index's portfolio assessment^9^, and because we considered that some of the ten public goals were more relevant in coral reefs than others, we considered both the ocean health index and some of its sub-components, namely ‘food provision/fisheries', ‘artisanal fisheries', ‘coastal livelihoods and economies', ‘sense of place', ‘biodiversity' and ‘coastal protection'. We used principal component analysis (Supplementary Fig. 13) and analysed the resulting correlation matrix to ensure that correlations among the ocean health sub-components we considered here were reasonably low. We did this because, like most statistical modelling techniques, boosted regression trees are sensitive to high multicollinearity among predictors^57^, so Pearson's correlation coefficient *r* should ideally be kept under 0.7. For all correlations among the ocean health index sub-components we considered, *r*<0.7 (range=(−0.46; 0.69); mean=0.23; median=0.27).

### Data management and quality control of fish data

*Data classification and data source effects*. The extent and quality of the data used in the study has only been possible by merging different data sets that have been collected and published independently (Supplementary Table 5). Merging these data required a set of sample qualifiers (for example, country, island and location), reclassification of each data set according to these qualifiers, and testing for potential data source effects, and potential temporal effects that could result from differences in the timing of data collection.

We defined country based on geopolitical units (for example, French Polynesia), which included multiple islands typically 100–1,000 km apart, and with different sampled locations on each island (for example, ocean-facing barrier reef) typically 10–100 km apart. Most countries comprised archipelagos or sets of islands and could easily be classified according to this scheme; however, for larger countries with extended reef systems (for example, Australia), a set of reefs (for example, Cairns) was classified as the island and a particular reef within that set (for example, Green Island Reef) as the location. This allowed us to keep a consistent definition of the spatial extent and resolution corresponding to each qualifier across data sets. Within each location, a sample typically corresponded to a site or a station where several replicates (transects or stationary point counts) were collected, across which we pooled the fish data for analysis. For the analysis, location was used as the sampling unit (corresponding to an average total sampled area of 2,760 m^2^, range 1,200–4,000 m^2^), which allowed us to minimize issues of spatial autocorrelation and random sampling error.

We tested for potential data source effects using countries and species that were sampled in multiple data sets. These countries included, for example, French Polynesia, New Caledonia, Tonga, Samoa. We compared the probability of presence of each species in each country, according to each data set, and tested for potential differences among data sets by using a permutational multivariate analysis of variance using distance matrices^58^ (permanova; function ‘adonis' in R package vegan). Similarly, for data sets with temporal replicates (for example, New Caledonia, Solomon Islands, Lord Howe Island), we tested for both seasonal and interannual differences in species' probabilities of occurrence using permanova.

*Missing fish data*. The PROCFish and WCS data sets included unavailable records for 91 species and 1,650 samples, and 175 species and 247 samples respectively, out of 241 species and 9,828 samples in the entire data set. For several species with incomplete sampling, some PROCFish and WCS locations fell beyond their geographic range, which thus limited the impact of missing data for such species. This resulted in 973 locations with all (potentially present) species sampled, and 103 locations with 15–59% species sampled. For such species, missing data represented on average 13% of all records (median 2%), which in some cases prevented model convergence; such species were thus not considered in further analyses (i.e., generalized linear mixed-effect models).

*Detectability models*. We ran detectability models before the occurrence models to assess whether sampled area (which differed among data sets) affected the detection of different species based on their body size or behaviour, and whether this effect varied across the geographical or correlate space (in which case detectability could have interfered with our models). Whereas detectability and occupancy can in theory be predicted simultaneously in occupancy models based on a joint probability distribution^59^, current modelling packages do not handle variable transect-level replication as is the case here; in such situations decoupling of processes is recommended (A. MacNeil, Australian Institute of Marine Science, Townsville, Australia; personal communication).

As a proxy for detectability, we calculated the proportion of replicate samples where each species was recorded at each location (*P*), given that it was present at that location. That is, at a given location, a species sighted on 1/4 of all transects (*P*=0.25) was deemed less detectable than a species sighted on 4/4 of all transects (*P*=1) this also depends on individual transect size, which we accounted for as an offset in the models. In the first model, we tested the idea that detectability would depend on body size and behaviour^21^ (i.e., mobility, schooling behaviour and water level). In the second model, we tested the idea that detectability would additionally vary depending on the geographical location, through the inclusion of two covariates: the region (Western Indian Ocean, Indo-Australian Archipelago or Western Pacific) and distance to the Coral Triangle. In the subsequent models, we tested whether detectability would additionally vary depending on the most important correlates of the presence models (distance to nearest land mass, total reef area within a 50-km radius, seasonality in sea surface temperature, human impact). We used hierarchical logistic regression (generalized linear mixed-effects with a binomial error distribution and a logit link) including random effects coding for the data set (to account for the non-independence of samples collected with the same methodology in a same data set) and for the genus nested within the family (to account for phylogenetic relationships among taxa).

We also tested whether potential behavioural avoidance of divers by fish could inflate the probability of recording false absences (missing a species when it is present) in heavily fished locations, particularly for targeted species that also tend to be large-bodied. We tested this hypothesis on a subset of our data for which distance-sampling observations were available. That is, fishes were also recorded beyond the 5-m wide transect on 3,630 of the GASPAR transects spanning a wide range of fishing pressure in New Caledonia, Fiji, Tonga and French Polynesia—these data have been published elsewhere^60^. Our hypothesis was that fishes recorded beyond 5 m are little affected by the presence of the diver and, in case of behavioural avoidance under fishing pressure, would only (or mostly) be recorded at such distances. We thus calculated the probability of recording false absences within 5 m as the proportion of transects where a species was only recorded beyond 5 m and, therefore, considered absent within 5 m. We compared the probability of recorded false absences along a gradient of fishing intensity (from 1: no or weak fishing to 5: intense fishing), both for all species and targeted/large ones.

### Modelling

*Occurrence models*. We used boosted regression trees^25^ (BRT) to identify the main correlates of occurrence patterns for each species within its range. We chose BRTs over other techniques because (i) they can handle a large number of predictors without over parameterizing; (ii) they are robust to moderate multicollinearity among predictors^57^ (Pearson's *r*∼0.7; in our case Pearson's *r*<0.7 for 95% of the among-predictor paired correlations), and (iii) they can fit non-linear relationships between response and predictor variables, as is often the case with ecological data^61^. We fitted BRT for each species using species presence/absence at each location as the response variable (*n*=906) and the range of correlates described above as predictors. We used a binomial (logistic) error distribution with a logit link. The total number of trees was determined by cross-validation^61^ and we set all other parameters to BRT default options (tree complexity of 3, learning rate of 0.01 and a bag fraction of 0.5) to make model outputs readily comparable among species. BRT outputs consisted of the cross-validated percent deviance explained in the response variable, percent contribution of each correlate to the deviance explained, and marginal plots of the partial effect of each correlate on species probability of occurrence^61^. We fitted BRT in R 3.0.1 (ref. 62) using the package {gbm} and the functions provided in Elith *et al*.^61^

*Influence of life-history traits on occurrence model outputs*. After excluding the species for which BRTs did not converge (*n*=32, 13.7% of all species, consisting of 15 infrequent species and 17 species not specifically associated with coral reef habitats), we used generalized linear mixed-effect models (GLMM) to analyse the outputs of the BRTs (that is, cross-validated percent deviance explained in the probability of the occurrence of each species and the relative contribution (%) of each correlate to the total deviance explained) as a function of a species life-history traits (for example, body size, range size, diet, mobility) and their interactions. We first summed the relative contributions across correlates related to the same hypothesis to calculate the relative contribution of each hypothesis (biogeography; area; energy; human pressure) to the deviance explained in species' occurrence patterns. The relative contribution of each of these four hypotheses, in addition to the total deviance explained, resulted in five response variables that we modelled using five separate GLMMs. Models included a random effect coding for genus nested within family as a partial control for phylogenetic non-independence among taxa^32^. Taxonomic hierarchies provide a valid proxy for phylogenetic relationships when molecular phylogenies are not available^63^, which was the case here. For each response variable, we assumed a Gaussian error distribution with a log link function and checked the normal distribution of model residuals using the normalized scores of standardized residual deviance^64^. We assessed GLMM performance using the marginal *R*^2^ (*R*_m_, variance explained by the fixed effects), and the conditional *R*^2^ (*R*_c_, variance explained by both the fixed and random effects) to provide an index of the model's goodness-of-fit^65^, Akaike's information criterion corrected for small sample sizes (AIC_c_) to provide an index of Kullback-Leibler information loss and corresponding weights (*w*AIC_c_) that assign relative strengths of evidence to the different competing models^66^. This information-theoretic approach offers a more robust method than standard regression techniques for testing alternative hypotheses because it uses a multimodel inference framework without arbitrary thresholds such as *P* values^67^. For each response variable, the first model sets included all individual life-history traits (for example, body size, range size, diet, mobility), in addition to the null (intercept-only) model. Among these models, we only included in the final model sets those for which *w*AIC_*c*_ was higher than for the null model (zero) as well as their paired linear combinations, with and without interactions. We fitted GLMM using the function lmer {lme4} in R 3.0.1 (ref. 62).

Based on the final model sets, we used the GLMM to predict the percent deviance explained in species occurrence patterns and the relative contributions (%) of the different hypotheses across the full range of life-history traits and their interactions. We used a model-averaging procedure where predictions from each model were weighted by its *w*AIC_*c*_ and summed across the model set^66^. Response surfaces were plotted in three-dimensional space using the function persp in R 3.0.1 (ref. 62).

*Null models*. To test the null hypothesis that the patterns we observed were not different from those expected by chance, we ran null models where we randomized the presences and absences of each species within its range. We then repeated the (i) BRT and (ii) GLMM analyses as described above. We applied a single randomization of the 241 species-specific BRT (to keep the time required to compute all models reasonable), thus corresponding to 241 species-specific null models.

*Relationship between range and body sizes*. We predicted geographical range size as a function of body size (that is, maximum adult total length) using separate GLMM with a Gaussian error distribution and a log link function, and other parameters as described above.

*Partial effects of occurrence correlates and mapping of global patterns*. We identified the strongest correlates of occurrence and plotted their partial effects (individual correlate effect, once the effect of other correlates had been accounted for) for each species. We then plotted the mean partial effects, averaged across species of three body size classes (≤15, 16–50,>50 cm), along with their 95% confidence intervals. For each body size class, we also plotted the mean partial effects for small-ranging species, defined as species within the first quartile of geographic range sizes. We tested for critical thresholds in these partial effects using the Davies test and, where present (*P*<0.05), identified their values based on a segmented linear regression^68^,^69^. We mapped a raster surface of the mean partial effect of human impact and temperature seasonality on the occurrence of large tropical reef fishes (>50 cm body size) across the Indo-Pacific using bilinear interpolation in ArcGIS 10.0.

## Additional information

**How to cite this article:** Mellin, C. *et al*. Humans and seasonal climate variability threaten large-bodied coral reef fish with small ranges. *Nat. Commun.* 7:10491 doi: 10.1038/ncomms10491 (2016).

## Supplementary Material

Supplementary Information Supplementary Figures 1-13, Supplementary Tables 1-6 and Supplementary References.

Supplementary Data 1 Study species and their biological traits, including maximum adult total length (Size), geographical range size (extent of occurrence; EOO), diet (FC: piscivore; HD: herbivoredetritivore feeding mainly on turf, micro-algae and detritus; HM: herbivores feeding mainly on large fleshy algae; IM: mobile invertebrate feeders; IS: sessile invertebrate feeders; OM: omnivores; PK: plankton feeders), home range(Sed: sedentary; Mob: mobile; VMob: very mobile), diel activity and schooling behaviour (Sol: solitary; Pair: paired; SmallG: small groups; MedG: medium groups; LargeG: large groups). Fish codes indicated in bold indicate largebodied, small-ranged fishes (Size = 50 cm and EOO < 90 × 106 km2).

## Figures and Tables

**Table 1:** Critical thresholds in the probability of occurrence of fish species of increasing body size in response to biogeography, energy, area-related correlates and human impact.

**Fish body size (cm)**	**Hypothesis**	**Correlate**	**Unit**	**Threshold (*****T*****)**	**Rate of change**	
					**Before** ***T***	**After** ***T***
<15	Biogeography	Dist2Land	km	494.1±13.0	−1.40	−0.08
<15	Area	ReefArea_50_	km^2^	1006.0±25.4	1.20	−1.91
<15	Area	ReefArea_50_	km^2^	1109.0±36.7	0.26	−0.93
<15	Energy	SST_sdev_	°C	3.4±0.1	1.53	0.00
<15	Human pressure	Human impact	—	36.3±0.8	0.00	1.10
15–50	Biogeography	Dist2Land	km	2868.0±56.7	0.03	0.71
15–50	Area	ReefArea_50_	km^2^	1021.0±30.3	1.05	−0.02
15–50	Energy	SST_sdev_	°C	1.2±0.05	−2.42	0.22
15–50	Human pressure	Human impact	—	30.6±0.6	−0.06	0.88
>50	Biogeography	Dist2Land	km	2771.0±42.7	0.17	1.35
>50	Area	ReefArea_50_	km^2^	956.0±24.8	0.95	0.03
>50	Energy	SST_sdev_	°C	1.2±0.0	−3.71	0.33
>50	Human pressure	Human impact	—	9.85±0.1	−2.53	−0.32

*Dist2Land*: distance to nearest land mass; *ReefArea*_*50*_: reef area; *SST*_*sdev*_: seasonal deviation (that is, seasonality) in sea surface temperature. Rate of change represents the slope of a linear relationship with all *x* axes rescaled to the [0; 1] interval to facilitate comparisons.

## References

[b1] Nogues-Bravo D., Araujo M. B., Romdal T. & Rahbek C. Scale effects and human impact on the elevational species richness gradients. Nature 453, 216–219 (2008). 18464741 10.1038/nature06812

[b2] Tittensor D. P. . Global patterns and predictors of marine biodiversity across taxa. Nature 466, 1098–1101 (2010). 20668450 10.1038/nature09329

[b3] Bellwood D. R. & Hughes T. Regional-scale assembly rules and biodiversity of coral reefs. Science 292, 1532–1534 (2001). 11375488 10.1126/science.1058635

[b4] MacArthur R. H. & Wilson E. O. The Theory of Island Biogeography Princeton University Press (1967).

[b5] Bellwood D. R. . Environmental and geometric constraints on Indo-Pacific coral reef biodiversity. Ecol. Lett. 8, 643–651 (2005).

[b6] Parravicini V. . Global patterns and predictors of tropical reef fish species richness. Ecography 36, 1254–1262 (2013).

[b7] Burke L. . Reefs at Risk Revisited World Resources Institute (2011).

[b8] Halpern B. S. . A global map of human impact on marine ecosystems. Science 319, 948–952 (2008). 18276889 10.1126/science.1149345

[b9] Halpern B. S. . An index to assess the health and benefits of the global ocean. Nature 488, 615–620 (2012). 22895186 10.1038/nature11397

[b10] Stuart-Smith R. D. . Integrating abundance and functional traits reveals new global hotspots of fish diversity. Nature 501, 539–542 (2013). 24067714 10.1038/nature12529

[b11] Mouillot D. . Rare species support vulnerable ecosystem functions in high-diversity ecosystems. PLoS Biol. 11, e1001569 (2014). 23723735 10.1371/journal.pbio.1001569 PMC3665844

[b12] Naeem S. . The functions of biological diversity in an age of extinction. Science 336, 1401–1406 (2012). 22700920 10.1126/science.1215855

[b13] Terribile L. C. . Richness patterns, species distributions and the principle of extreme deconstruction. Global Ecol. Biogeogr. 18, 123–136 (2009).

[b14] Cheung W. W. . Signature of ocean warming in global fisheries catch. Nature 497, 365–368 (2013). 23676754 10.1038/nature12156

[b15] McClanahan T. & Humphries A. Differential and slow life-history responses of fishes to coral reef closures. Mar. Ecol. Prog. Ser. 469, 121–131 (2012).

[b16] McCauley D. J. . Marine defaunation: animal loss in the global ocean. Science 347, 1255641 (2015). 25593191 10.1126/science.1255641

[b17] Worm B. & Tittensor D. P. Range contraction in large pelagic predators. Proc. Natl Acad. Sci. USA 108, 11942–11947 (2011). 21693644 10.1073/pnas.1102353108 PMC3141942

[b18] Harnik P. G., Simpson C. & Payne J. L. Long-term differences in extinction risk among the seven forms of rarity. Proc. Biol. Sci. 279, 4969–4976 (2012). 23097507 10.1098/rspb.2012.1902 PMC3497235

[b19] Graham N. A. . Extinction vulnerability of coral reef fishes. Ecol. Lett. 14, 341–348 (2011). 21320260 10.1111/j.1461-0248.2011.01592.x PMC3627313

[b20] Hughes T. P. . Double jeopardy and global extinction risk in corals and reef fishes. Curr. Biol. 24, 2946–2951 (2014). 25454782 10.1016/j.cub.2014.10.037

[b21] Kulbicki M. . *Major Coral Reef Fish Species of the South Pacific with Basic Information on their Biology and Ecology. CRISP-IRD Report.* 107 (Secretariat of the Pacific Community, Noumea, 2011).

[b22] Froese R. & Pauly D. FishBase World Wide Web electronic publication www.fishbase.org version (11/2014 (2014).

[b23] Gaston K. J. & Fuller R. A. The sizes of species' geographic ranges. J. Appl. Ecol. 46, 1–9 (2009).

[b24] Kulbicki M. . Global biogeography of reef fishes: a hierarchical quantitative delineation of regions. PLoS ONE 8, e81847 (2013). 24386083 10.1371/journal.pone.0081847 PMC3875412

[b25] Friedman J. H. Greedy function approximation: a gradient boosting machine. Ann. Stat. 29, 1189–1232 (2001).

[b26] Wiens J. J. & Donoghue M. J. Historical biogeography, ecology and species richness. Trends Ecol. Evol. 19, 639–644 (2004). 16701326 10.1016/j.tree.2004.09.011

[b27] Mora C. in The Ecology of Fishes on Coral Reefs ed. Mora C. Academic Press (2014).

[b28] Mellin C. . Reef size and isolation determine the temporal stability of coral reef fish populations. Ecology 91, 3138–3145 (2010). 21141175 10.1890/10-0267.1

[b29] Brown J. H. On the relationship between abundance and distribution of species. Am. Nat. 124, 255–279 (1984).

[b30] Andersen K. & Beyer J. Asymptotic size determines species abundance in the marine size spectrum. Am. Nat. 168, 54–61 (2006). 16685635 10.1086/504849

[b31] Jennings S. & Mackinson S. Abundance-body mass relationships in size-structured food webs. Ecol. Lett. 6, 971–974 (2003).

[b32] Luiz O. J. . Adult and larval traits as determinants of geographic range size among tropical reef fishes. Proc. Natl Acad. Sci. USA 110, 16498–16502 (2013). 24065830 10.1073/pnas.1304074110 PMC3799316

[b33] Edgar G. J. . Global conservation outcomes depend on marine protected areas with five key features. Nature 506, 216 (2014). 24499817 10.1038/nature13022

[b34] Graham N. A. J. . Size-spectra as indicators of the effects of fishing on coral reef fish assemblages. Coral Reefs 24, 118–124 (2005).

[b35] McClanahan T. R. . Toward pristine biomass: reef fish recovery in coral reef marine protected areas in Kenya. Ecol. Appl. 17, 1055–1067 (2007). 17555218 10.1890/06-1450

[b36] Wilson S. . Habitat degradation and fishing effects on the size structure of coral reef fish communities. Ecol. Appl. 20, 442–451 (2010). 20405798 10.1890/08-2205.1

[b37] Atkinson D. & Sibly R. M. Why are organisms usually bigger in colder environments? Making sense of a life history puzzle. Trends Ecol. Evol. 12, 235–239 (1997). 21238056 10.1016/s0169-5347(97)01058-6

[b38] Poloczanska E. S. . Global imprint of climate change on marine life. Nature Clim. Change 3, 919–925 (2013).

[b39] Przeslawski R. . A review and meta-analysis of the effects of multiple abiotic stressors on marine embryos and larvae. Glob. Change Biol. 21, 2122–2140 (2015). 10.1111/gcb.12833 25488061

[b40] Burrows M. T. . The pace of shifting climate in marine and terrestrial ecosystems. Science 334, 652–655 (2011). 22053045 10.1126/science.1210288

[b41] Samoilys M. A. Periodicity of spawning aggregations of coral trout Plectropomus leopardus (Pisces: Serranidae) on the northern Great Barrier Reef. Mar. Ecol. Prog. Ser. 160, 149–159 (1997).

[b42] Garcia Molinos J. . Climate velocity and the future global redistribution of marine biodiversity. Nat. Clim. Change 21, 117–129 (2015).

[b43] Stuart-Smith R. D. . Thermal biases and vulnerability to warming in the world's marine fauna. Nature 528, 88–92 (2015). 26560025 10.1038/nature16144

[b44] Cai W. . Increased frequency of extreme La Nin˜a events under greenhouse warming. Nat. Clim. Change 5, 132–137 (2015).

[b45] Rahmstorf S. & Coumou D. Increase of extreme events in a warming world. Proc. Natl Acad. Sci. USA 108, 17905–17909 (2011). 22025683 10.1073/pnas.1101766108 PMC3207670

[b46] Hoegh-Guldberg O. . Coral reefs under rapid climate change and ocean acidification. Science 318, 1737–1742 (2007). 18079392 10.1126/science.1152509

[b47] Knutson T. R. . Tropical cyclones and climate change. Nat. Geosci. 3, 157–163 (2010).

[b48] Hoegh-Guldberg O. & Bruno J. F. The impact of climate change on the world's marine ecosystems. Science 328, 1523–1528 (2010). 20558709 10.1126/science.1189930

[b49] Mace G. M. . Quantification of extinction risk: IUCN's system for classifying threatened species. Conserv. Biol. 22, 1424–1442 (2008). 18847444 10.1111/j.1523-1739.2008.01044.x

[b50] Bellwood D. R. . Confronting the coral reef crisis. Nature 429, 827–833 (2004). 15215854 10.1038/nature02691

[b51] D'agata S. . Human-mediated loss of phylogenetic and functional diversity in coral reef fishes. Curr. Biol. 24, 555–560 (2014). 24560574 10.1016/j.cub.2014.01.049

[b52] Halford A. R. & Thompson A. A. Visual Census Surveys of Reef Fish. Long term monitoring of the Great Barrier Reef Standard Operational Procedure Number 3 Australian Institute of Marine Science (1996).

[b53] Ayotte P. . Coral Reef Ecosystem Division Standard Operating Procedures: Data Collection for Rapid Ecological Assessment Fish Surveys Pacific Islands Fisheries Science Center (2011).

[b54] Mora C. . Global human footprint on the linkage between biodiversity and ecosystem functioning in reef fishes. PLoS Biol. 9, e1000606 (2011). 21483714 10.1371/journal.pbio.1000606 PMC3071368

[b55] Behrenfeld M. J. . Climate-driven trends in contemporary ocean productivity. Nature 444, 752–755 (2006). 17151666 10.1038/nature05317

[b56] Hijmans R. J. & Graham C. H. The ability of climate envelope models to predict the effect of climate change on species distributions. Glob. Change Biol. 12, 2272–2281 (2006).

[b57] Dormann C. F. . Collinearity: a review of methods to deal with it and a simulation study evaluating their performance. Ecography 36, 27–46 (2013).

[b58] Anderson M. J. A new method for non-parametric multivariate analysis of variance. Aust. J. Ecol. 26, 32–46 (2001).

[b59] MacKenzie D. I. . Occupancy Estimation and Modeling: Inferring Patterns and Dynamics of Species Occurrence Elsevier (2006).

[b60] Kulbicki M. How the acquired behaviour of commercial reef fishes may influence the results obtained from visual censuses. J. Exp. Mar. Biol. Ecol 222, 11–30 (1998).

[b61] Elith J. . A working guide to boosted trees. J. Anim. Ecol. 77, 802–813 (2008). 18397250 10.1111/j.1365-2656.2008.01390.x

[b62] R Development Core Team, R: A language and environment for statistical computing (R Foundation for Statistical Computing, Vienna, Austria. ISBN 3-900051-07-0, URL http://www.R-project.org/ (2013).

[b63] Ricotta C. . Computing diversity from dated phylogenies and taxonomic hierarchies: does it make a difference to the conclusions? Oecologia 170, 501–506 (2012). 22526936 10.1007/s00442-012-2318-8

[b64] Breslow N. E. Generalized linear models: checking assumptions and strengthening conclusions. J. Stat. Appl. 8, 23–41 (1996).

[b65] Nakagawa S. & Schielzeth H. A general and simple method for obtaining *R*^2^ from generalized linear mixed-effects models. Methods Ecol. Evol. 4, 133–142 (2013).

[b66] Burnham K. P. & Anderson D. R. Model Selection and Multimodel Inference: A Practical Information Theoretic Approach 2nd ed Springer-Verlag (2002).

[b67] Burnham K. P. . AIC model selection and multimodel inference in behavioral ecology: some background, observations, and comparisons. Behav. Ecol. Sociobiol. 65, 23–35 (2011).

[b68] Muggeo V. M. R. Estimating regression models with unknown break-points. Stat. Med. 22, 3055–3071 (2003). 12973787 10.1002/sim.1545

[b69] Muggeo V. M. R. Segmented: an R package to fit regression models with broken-line relationships. R News 8, 20–25 (2008).

