# Supplementary material for: Humans and seasonal climate variability threaten large-bodied coral reef fish with small ranges
Source: Nat Commun. 2016 Feb 3;7:10491. doi: 10.1038/ncomms10491 (PMC4742806; doi:10.1038/ncomms10491)
Supplement: Supplementary Information — Supplementary Figures 1-13, Supplementary Tables 1-6 and Supplementary References. [file ncomms10491-s1.pdf]

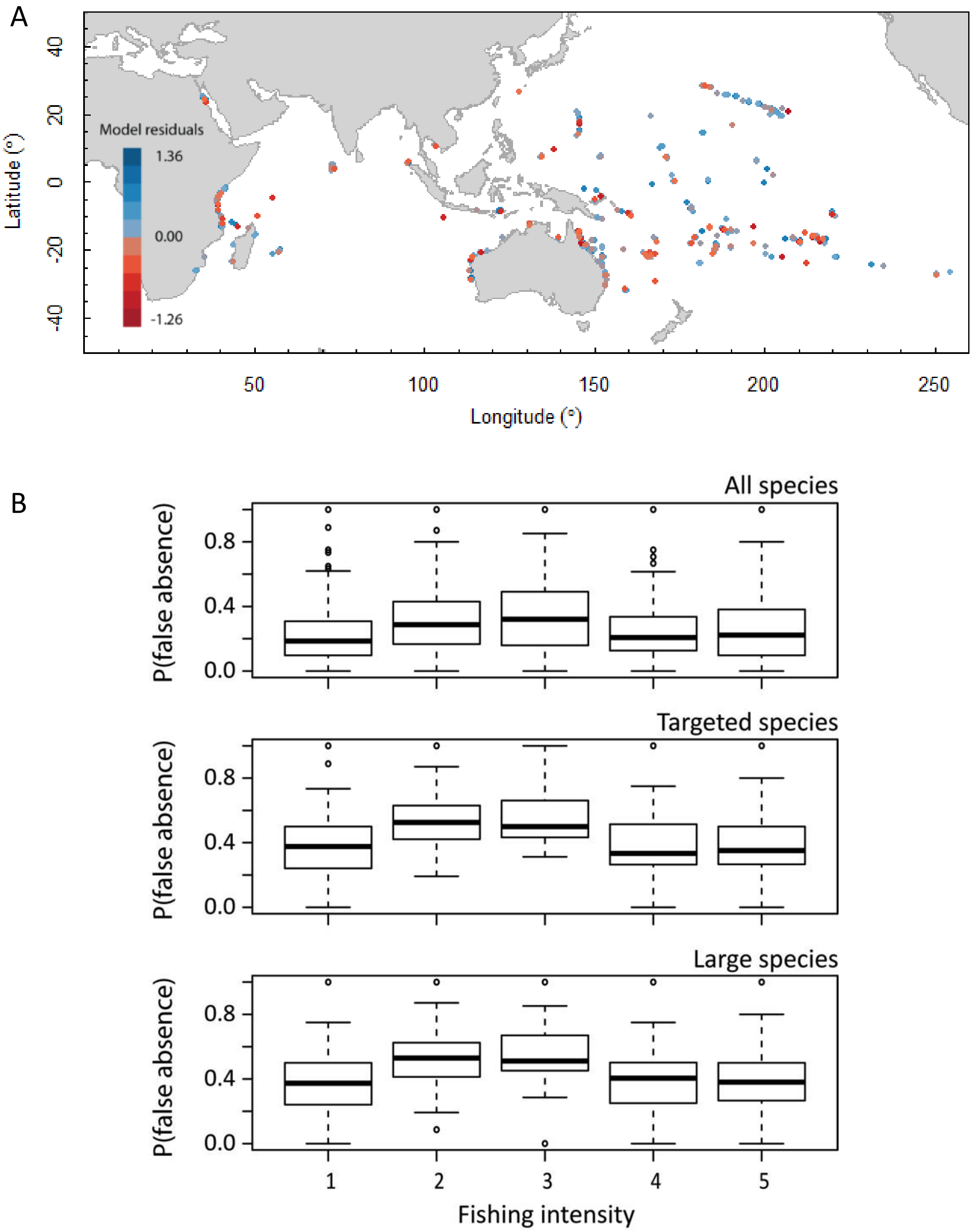

Supplementary Figure 1. Residuals of detectability models (A) accounting for fish body size and behaviour (and including sampled area as an offset) showing no spatial pattern across the study area, and (B) showing no effect of fishing intensity on the probability of recording false absences, either for all species or targeted/large ones.

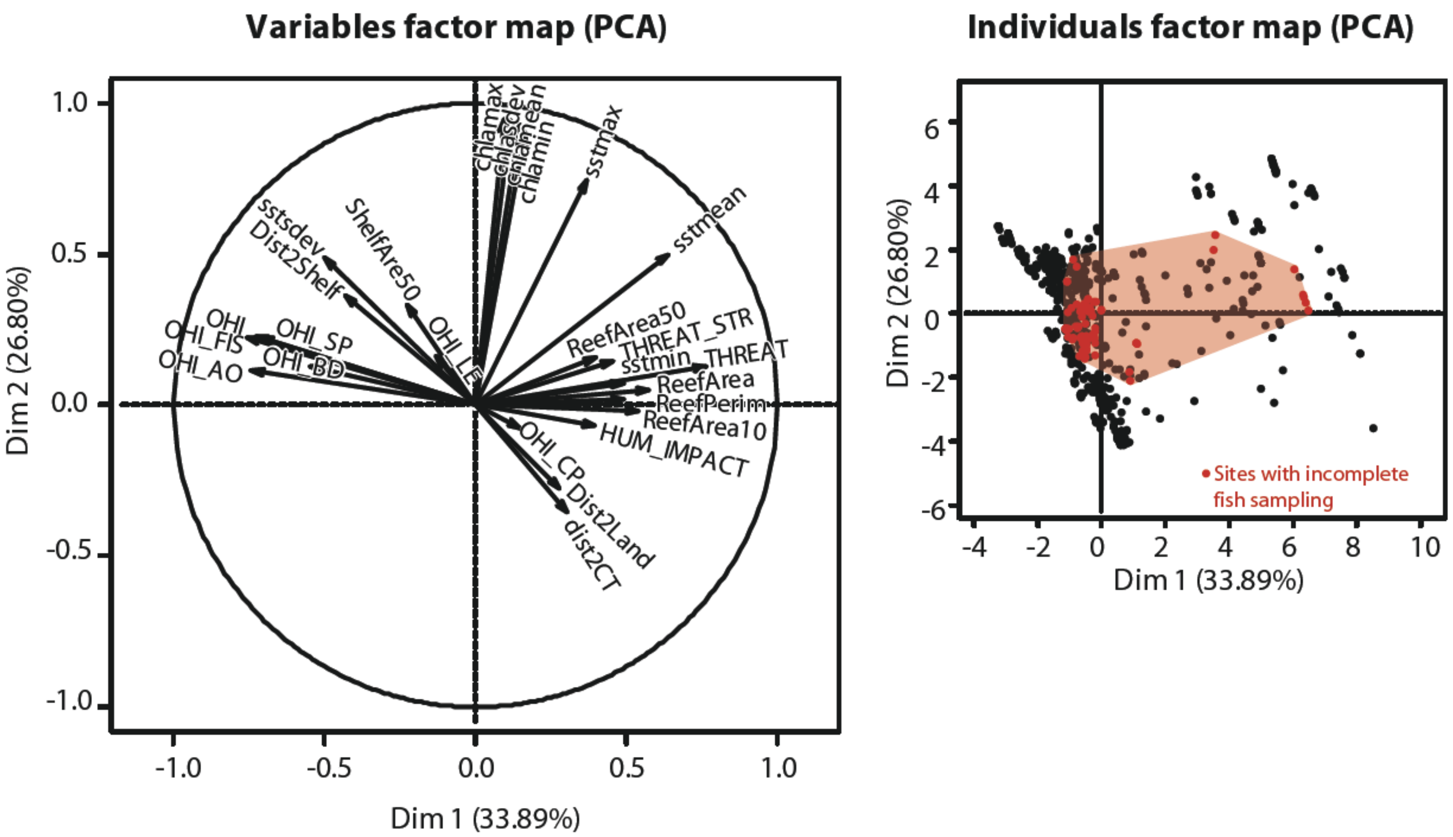

Supplementary Figure 2. Principal component analysis including the correlates (left; see Supplementary Table 6 for correlate codes) and the projection of sampled sites onto the correlate ordination plan (right), indicating that sites with some missing data (i.e. incomplete fish sampling, indicated in red) are evenly distributed across the correlate space.

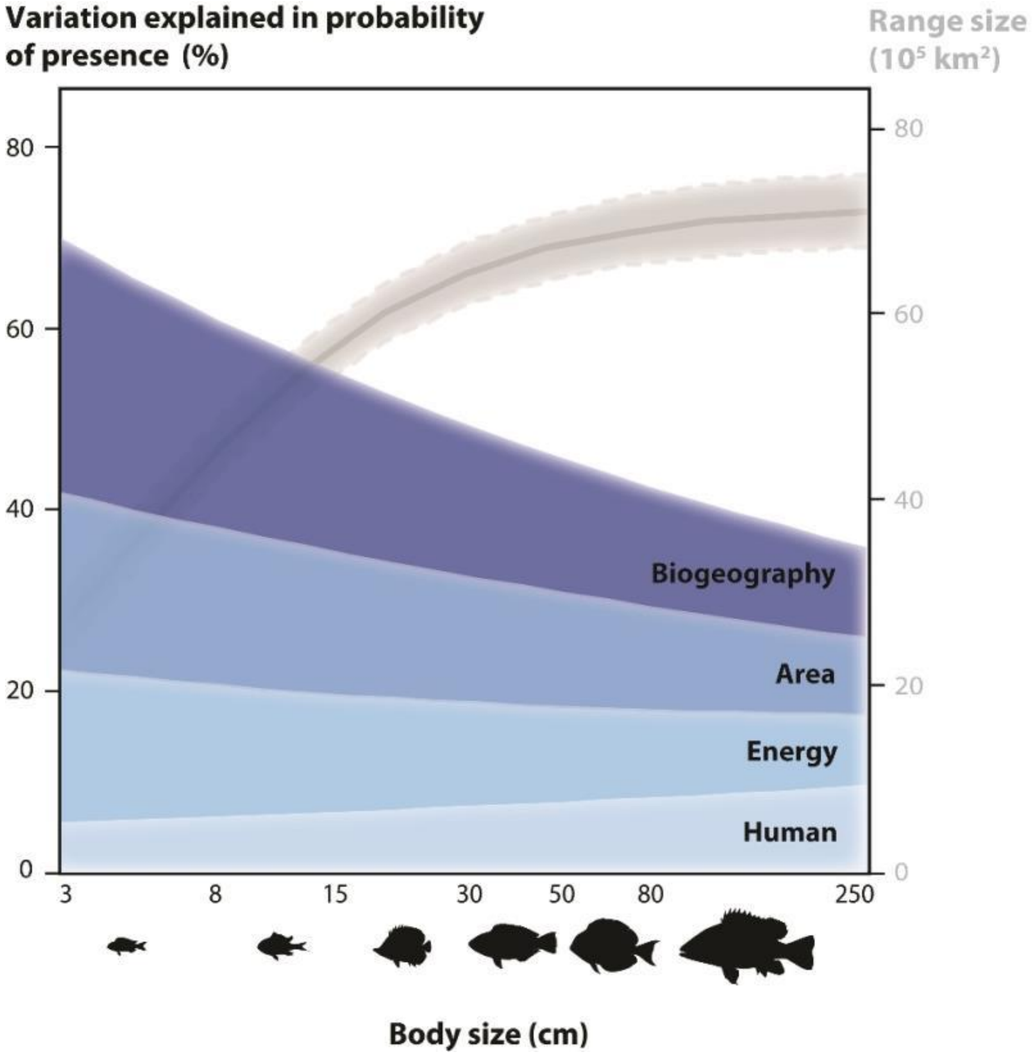

Supplementary Figure 3. Percent variation explained in species' probability of presence according to biogeography, energy, area or human pressure (blue envelopes, left *y*-axis) as a function of body size (maximum adult total length, log scale). The relationship between geographical range size and body size, modelled separately, is indicated by the grey envelope (mean and 95 % confidence interval) and corresponds to the right *y*-axis. Fish silhouettes illustrate characteristic species of each body size class (from left to right: *Chrysiptera rollandi*; *Chromis viridis*; *Chaetodon ulietensis*; *Scarus schlegeli*; *Acanthurus dussumieri*; *Lutjanus argentimaculatus*); their size is scaled to the log maximum adult total length.

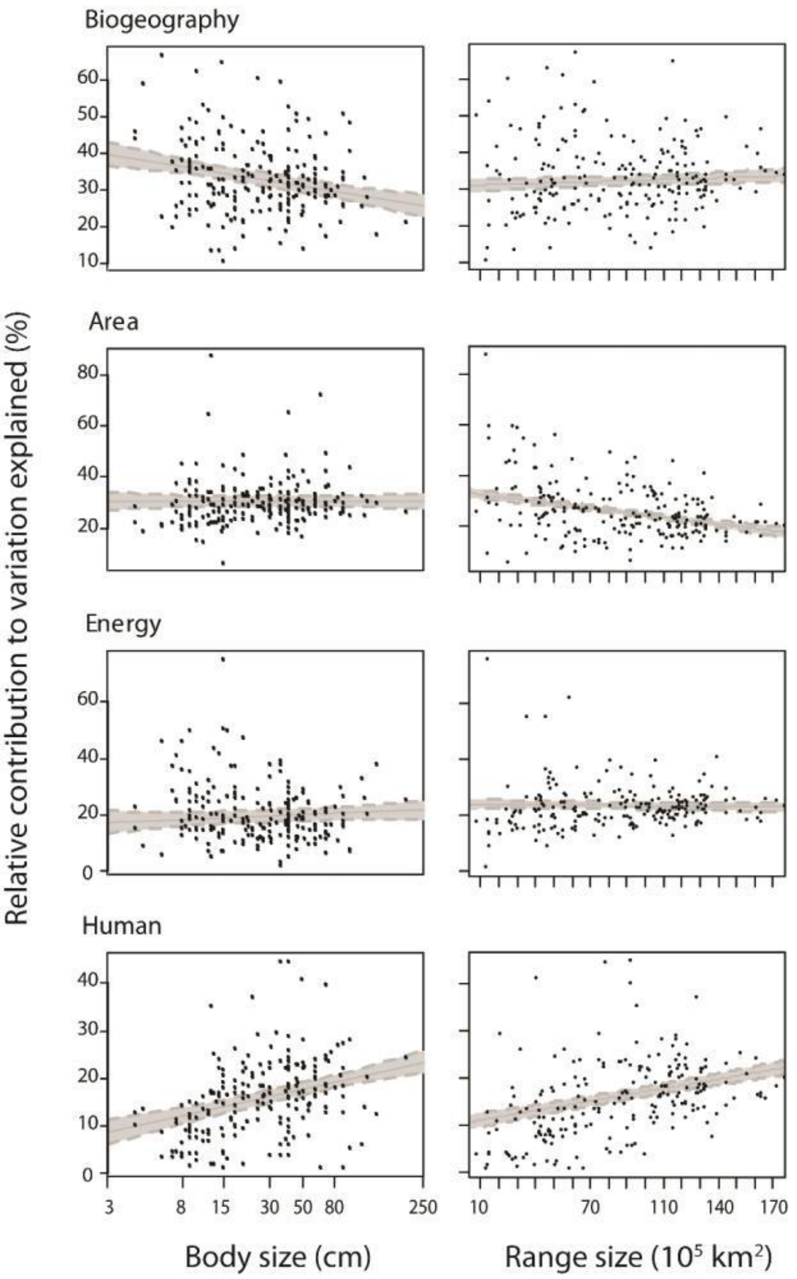

Supplementary Figure 4. Relative contribution (%) of biogeography, area, energy and human pressure correlates to the total variation explained in fish occurrence patterns (cross-validated deviance) as a function of maximum adult total length (*body size*; left) and geographical extent of occurrence (*range size*; right). Grey regression lines and envelopes (95 % confidence interval) show the linear fits (predictions) given by mixed-effect models with dots indicating individual observations.

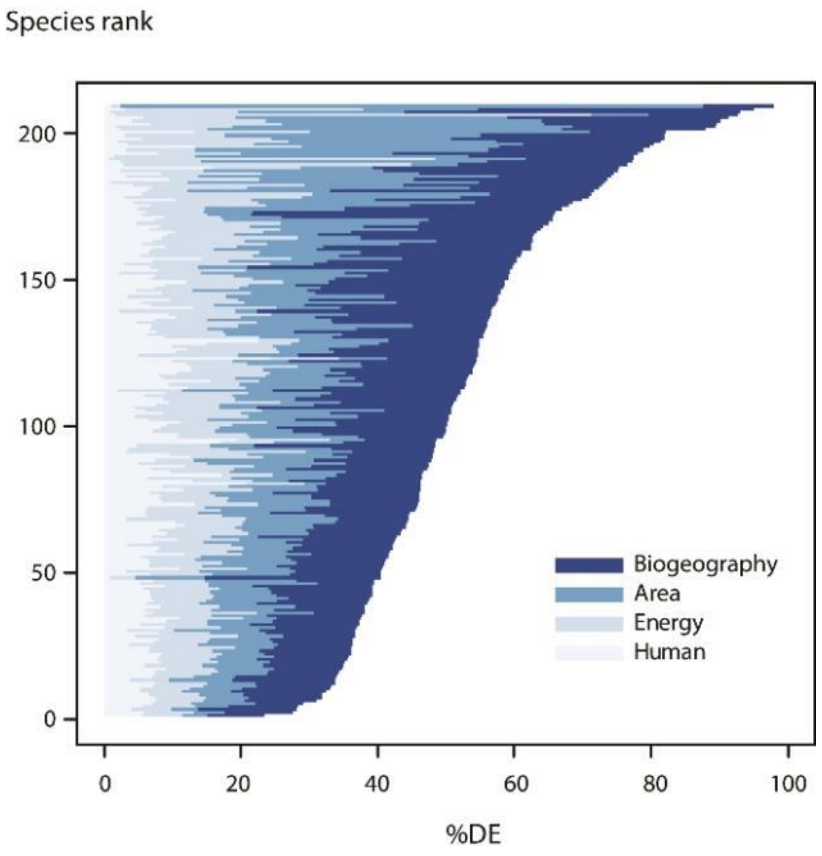

Supplementary Figure 5. Percent variation explained (cross-validated deviance, *%DE*) in the probability of presence of each species and relative contribution of each group of correlates estimated using boosted regression trees. Species are ranked by increasing *%DE*.

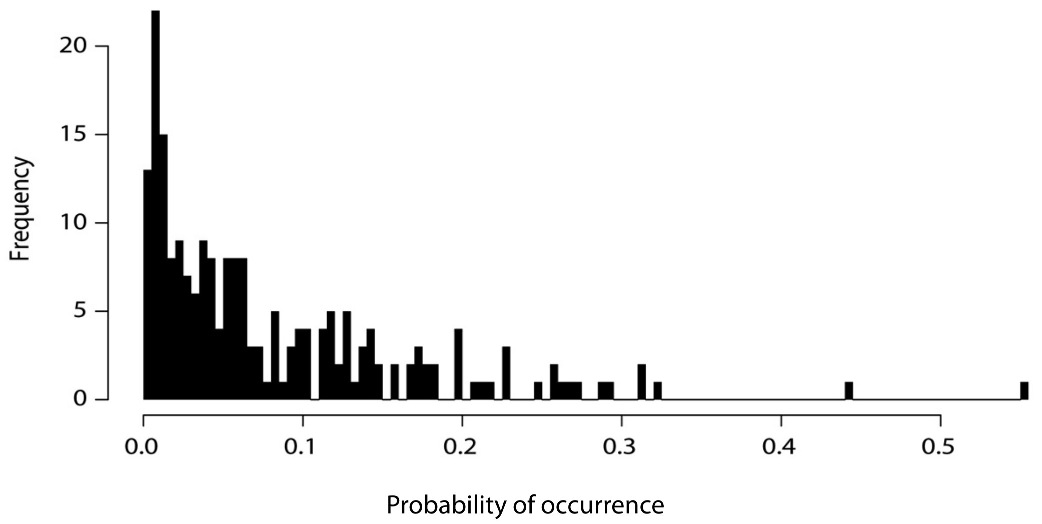

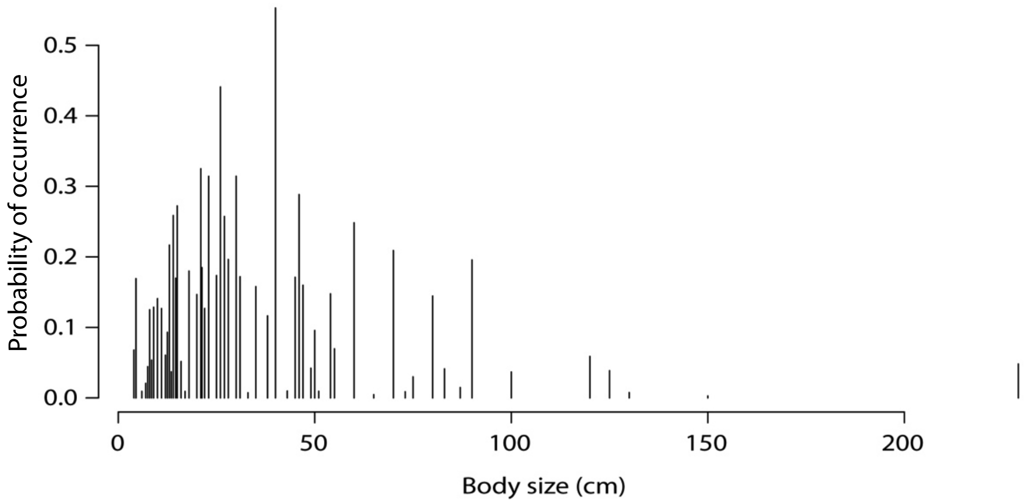

Supplementary Figure 6. Distribution of the probability of occurrence (ratio of sites where a species is present) (top panel) and relationship with maximum individual total length (body size, in cm) (bottom panel).

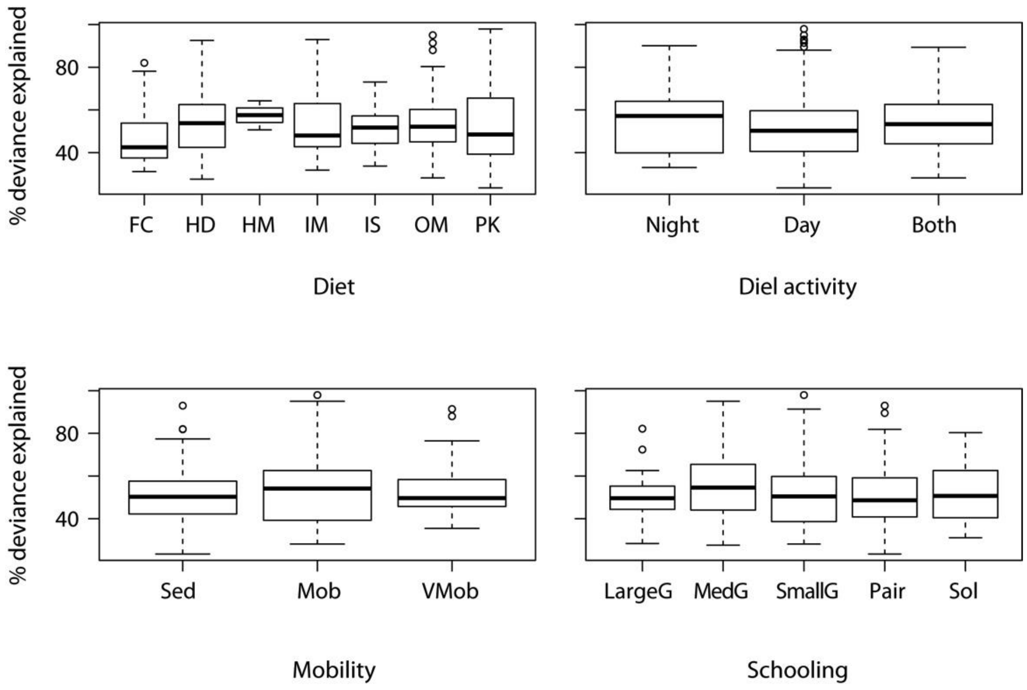

Supplementary Figure 7. Percent variation explained (cross-validated deviance) in the probability of presence of each species as a function of species traits including diet, diel activity, mobility and schooling behaviour.

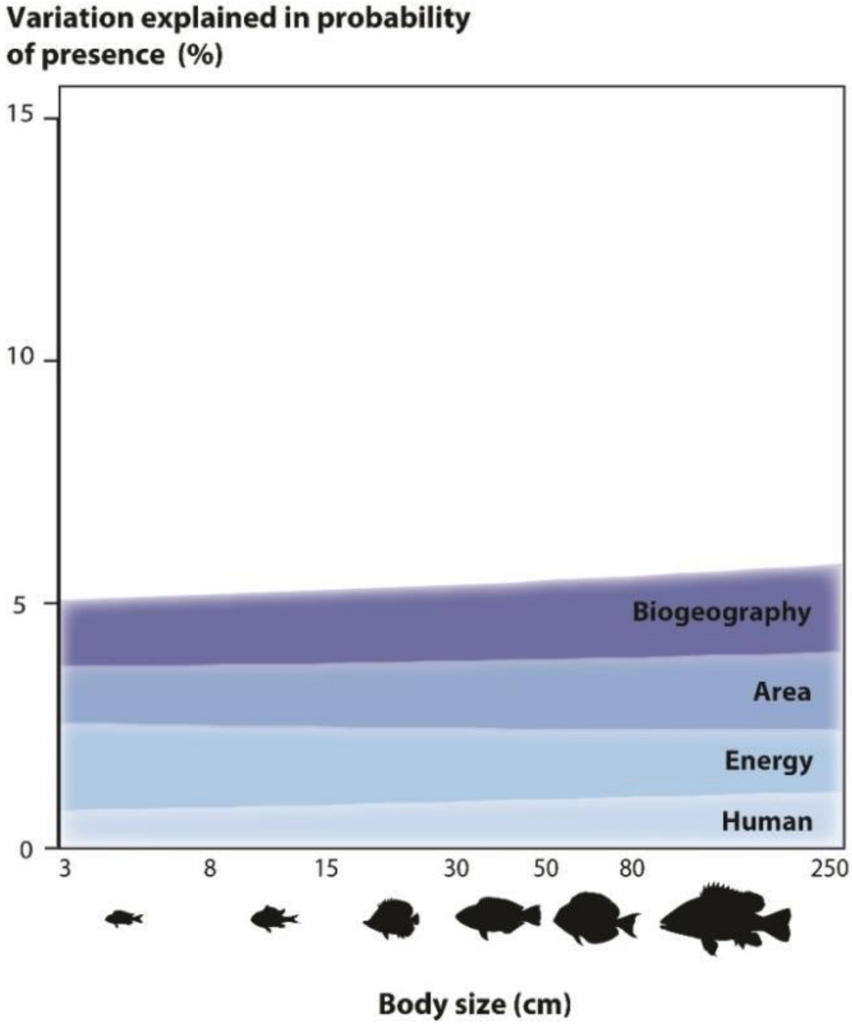

Supplementary Figure 8. Results of null models: percent variation explained in species' probability of presence according to biogeography, energy, area or human pressure as a function of body size (maximum adult total length, log scale). Fish silhouettes illustrate characteristic species of each body size class (from left to right: *Chrysiptera rollandi*; *Chromis viridis*; *Chaetodon ulietensis*; *Scarus schlegeli*; *Acanthurus dussumieri*; *Lutjanus argentimaculatus*); their size is scaled to the log maximum adult total length.

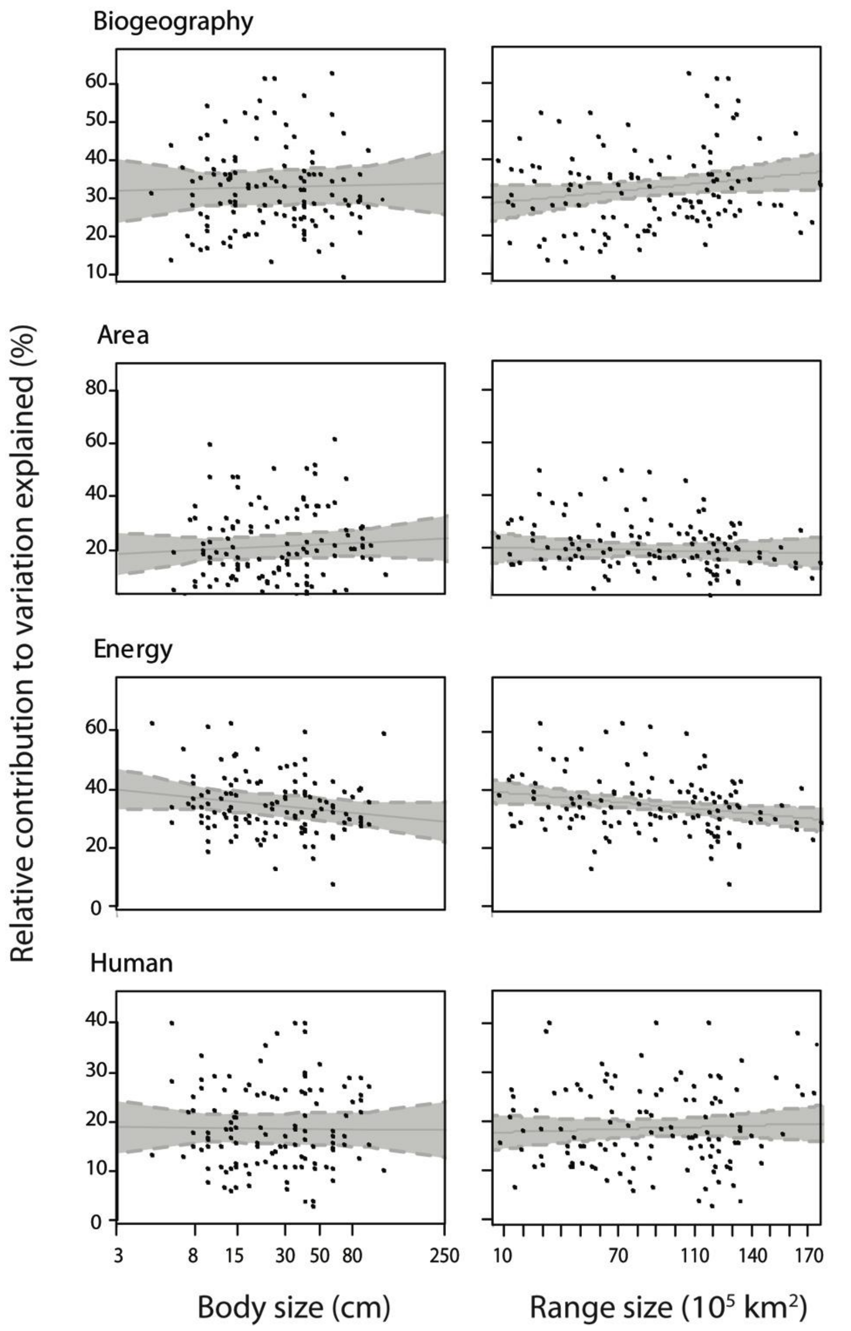

Supplementary Figure 9. Results of null models: Relative contribution (%) of biogeography, area, energy and human pressure correlates to the total variation explained in fish occurrence patterns (cross-validated deviance) as a function of maximum adult total length (*body size*; left) and geographical extent of occurrence (*range size*; right). Grey regression lines and envelopes (95 % confidence interval) show the linear fits (predictions) given by mixed-effect models with dots indicating individual observations.

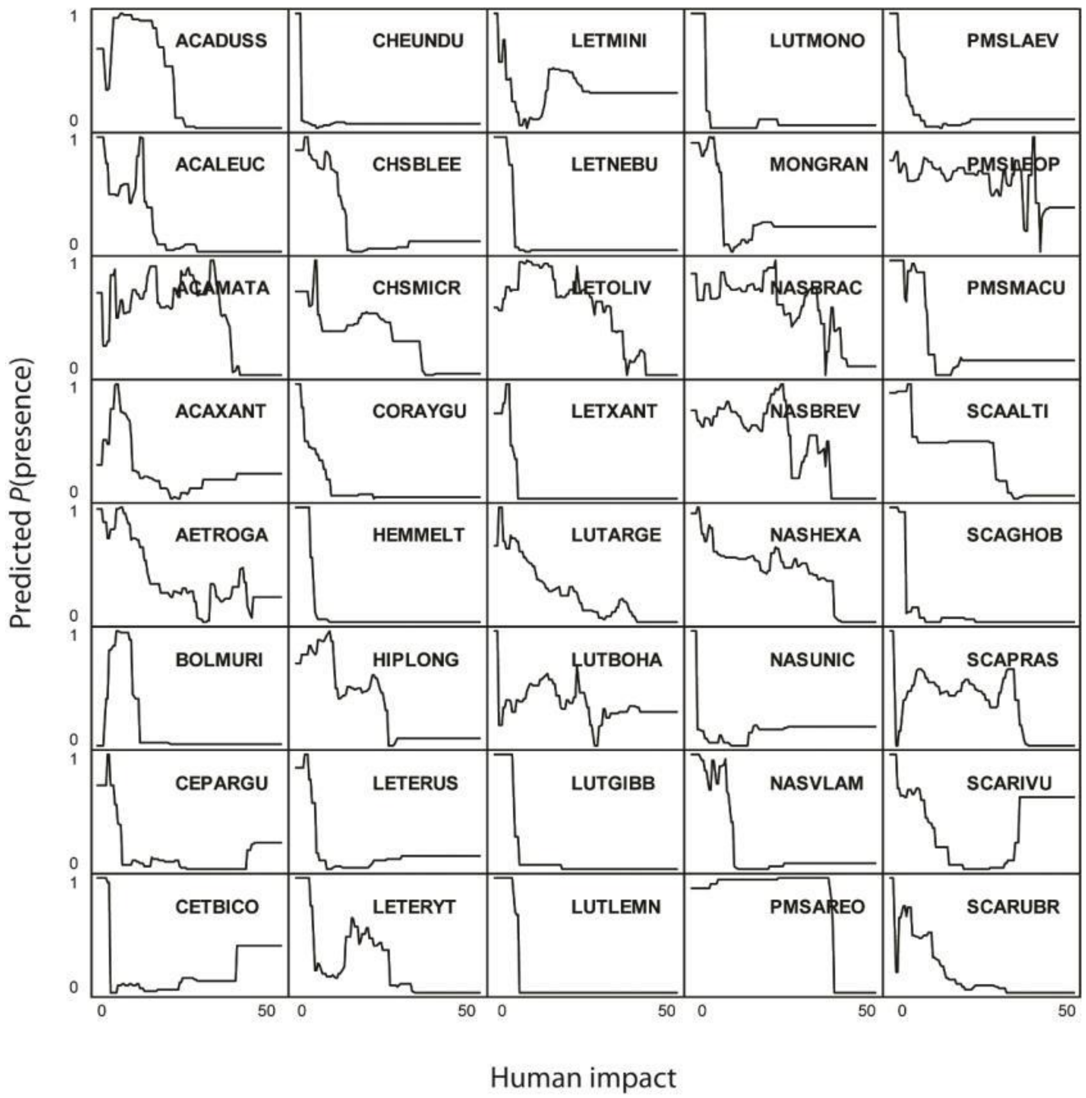

Supplementary Figure 10. Predicted probability of presence [*P* (presence)] of larger-bodied species (maximum adult total length > 50 cm) in response to human impact (range 0-50 as for the sampled reefs) estimated using boosted regression trees. See Supplementary Data 1 for fish codes.

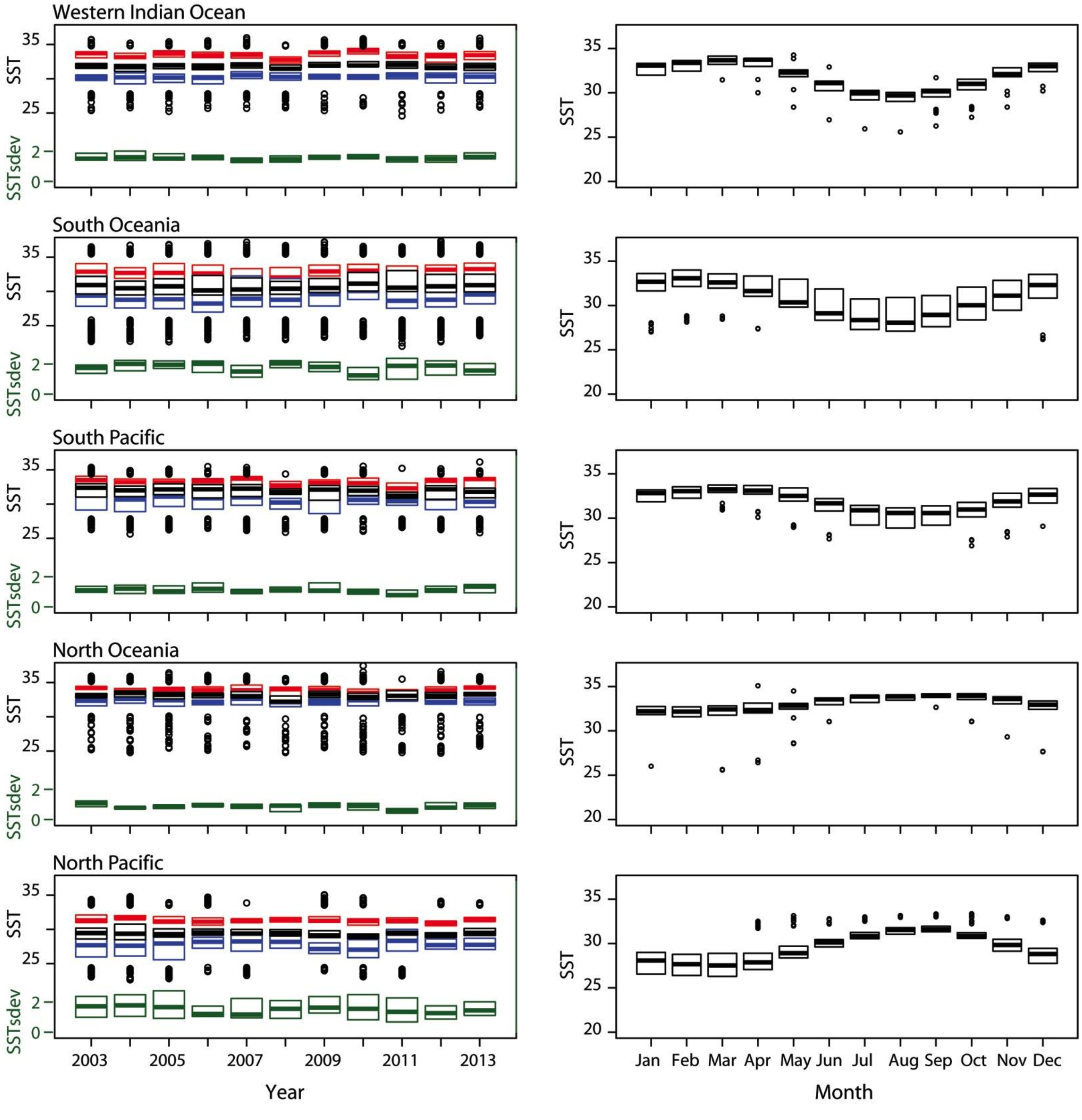

Supplementary Figure 11. Time series of sea surface temperature (SST, °C) and seasonal deviation in SST (SSTsdev, °C) recorded across the study area between 2003 and 2013. Yearly time series (left panel) show mean summer temperature (red), mean winter temperature (blue), mean annual temperature (black) and seasonality in temperature (green). Local extremes (i.e., 1° grid cells with a temperature outside of the 2nd — 98th percentile range, defined across all years considered) are represented by dots. Monthly time series (right) show monthly (climatological) SST averages. Top and bottom sides of the boxes show the first and third quartiles across all grid cells, the thick horizontal line in between shows the median. Data were extracted from MODIS Aqua monthly climatology at a 9-km resolution. A temporal trend was evident in South Oceania (increase in mean winter SST by 0.06 °C y^{-1}, increase in mean summer SST by

0.03 °C y^{-1}, increase in mean annual SST by 0.05 °C y^{-1} and decrease in seasonality by 0.01 °C y^{-1}; $p < 0.001$; linear model). The magnitude of negative temperature extremes increased by 0.11°C y^{-1} in the South Pacific and that of positive extremes increased by 0.23°C y^{-1} in the North Pacific ($p < 0.001$; linear model).

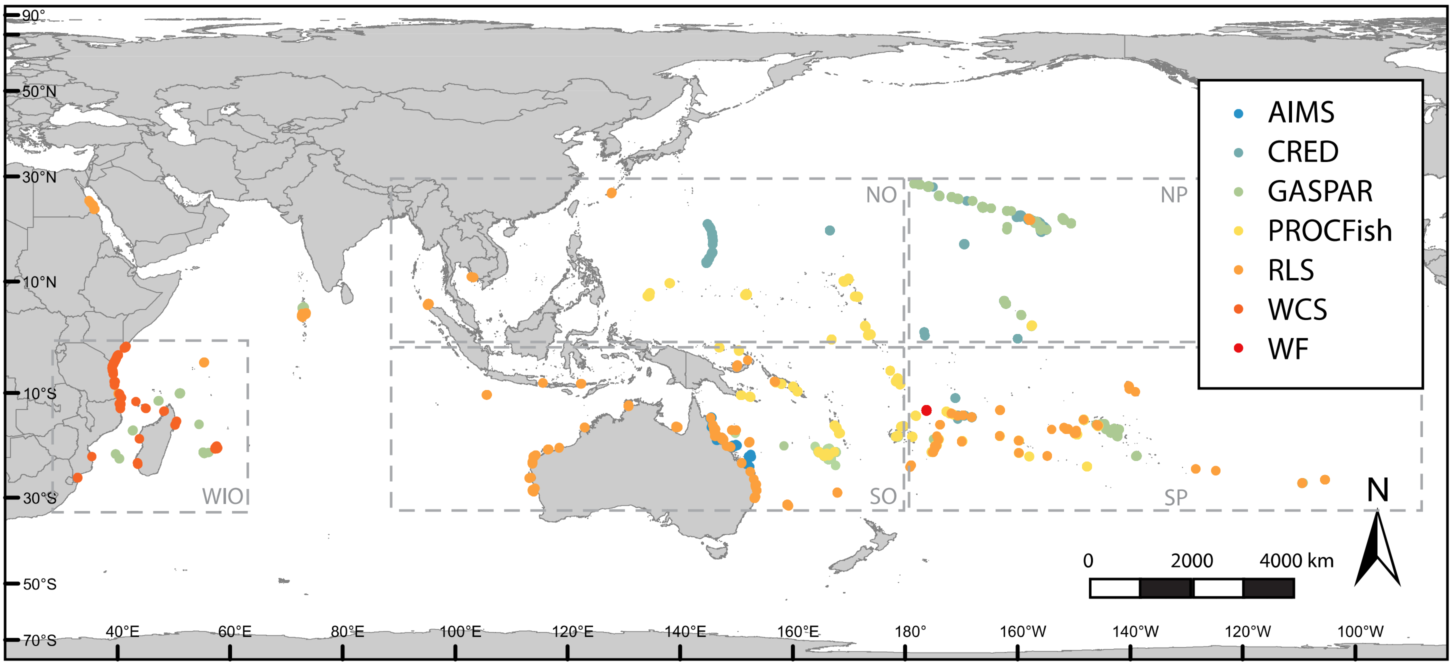

Supplementary Figure 12. Survey locations across the Indo Pacific for each individual dataset, with AIMS: Australian Institute of Marine Science; CRED: Coral Reef Ecosystem Division, University of Hawaii; GASPAR: General Approach to SPecies-Abundance Relationships; PROCFish: Pacific Regional Oceanic and Coastal Fisheries development programme, Secretariat of the Pacific Community (SPC); RLS: Reef Life Survey; WCS: Wildlife Conservation Society; WF: Wallis and Futuna. Grey dashed boxes show regions boundaries considered in time series of sea surface temperature, with WIO: Western Indian Ocean, NO: North Oceania, SO: South Oceania, NP: North Pacific, SP: South Pacific.

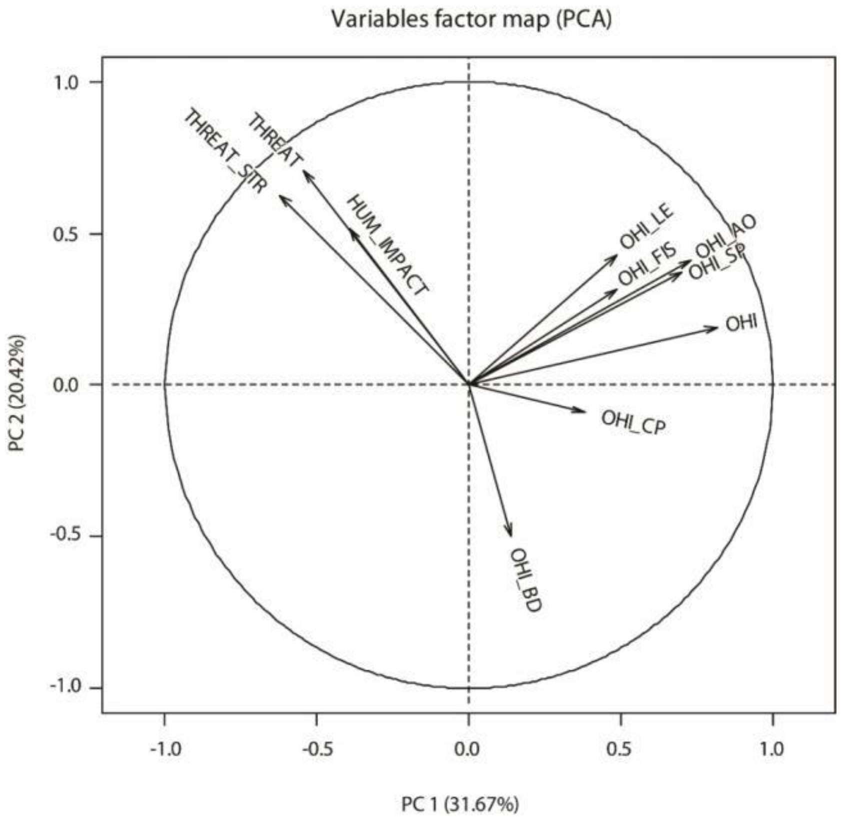

Supplementary Figure 13. Principal component analysis (PCA) of human pressure. PC1 and PC2 represent the first two axes of the PCA. See Supplementary Table 6 for variable code and description.

Supplementary Table 1 Results of the hierarchical logistic regressions of the proportion of transects on which a species was sighted at a given location. *BodySize* refers to the maximum adult total length (in cm). *Behaviour* encompasses mobility, schooling behaviour and water level (factors). See Supplementary Table 2 for other variable codes. Shown are the number of parameters (*k*), the log-likelihood (LogL), Akaike's information criterion corrected for small sample sizes (AIC_c) and AIC_c weights ($w\ AIC_c$) as well as the per cent deviance explained in each response variable (DE). In each model set, models are ordered by decreasing wAICc

**Model#**	**Model**	***k***	**LogL**	**AIC_c**	**$wAIC_c$**	**DE**
**1**	P ~ BodySize + Behaviour + offset (SampledArea)	16	-15966	31963.91	0.939	37.01
**2**	P ~ BodySize + Behaviour + region + Dist2CT + offset (SampledArea)	19	-15965.7	31969.37	0.061	37.01
**3**	P ~ BodySize + Behaviour + Dist2Land + ReefArea50 + sstsdev + HUM_IMPACT + offset (SampledArea)	20	-19860.6	39761.19	0.000	21.64
**4**	P ~ 1	3	-25345.3	50696.57	0.000	0.00

Supplementary Table 2. Results of the generalized linear mixed-effect models predicting the total deviance explained in fish occurrence patterns by boosted regression trees (DE) and the relative contribution of biogeography-, area- and energy-related correlates and human pressure as a function of maximum adult total length (*BodySize*, in cm) and extent of occurrence (*RangeSize*, in 10^6 km^2). Models included a random effect coding for the genus nested within the family (1|Family/Genus) to account for phylogenetic relationships among taxa. Shown are the number of parameters (*k*), the log-likelihood (*LogL*), Akaike's information criterion corrected for small sample sizes (AIC_c) and AIC_c weights ($wAIC_c$), the marginal R^2 (R_m) and conditional R2 (R_c). In each model set, models are ordered by decreasing $wAIC_c$, with the best model based on $wAIC_c$ indicated in bold.

Model	*k*	LogL	AIC_c	$wAIC_c$	R_m	R_c
**DE ~ BodySize * RangeSize**	7	-247.03	508.63	**0.822**	33.37	46.07
DE ~ BodySize + RangeSize	6	-249.63	511.68	0.178	33.62	46.13
DE ~ 1	3	-447.10	900.32	0.000	0.00	31.36
**BIOGEOGRAPHY ~ BodySize + RangeSize**	6	-766.92	1546.25	**0.789**	8.00	16.14
BIOGEOGRAPHY ~ BodySize * RangeSize	7	-767.24	1549.04	0.196	6.87	14.70
BIOGEOGRAPHY ~ 1	3	-774.11	1554.33	0.014	0.00	11.00
**AREA ~ BodySize + RangeSize**	6	-334.85	684.25	**1.000**	13.37	19.64
AREA ~ BodySize * RangeSize	7	-343.99	700.40	0.000	10.92	17.93
AREA ~ 1	3	-459.80	925.71	0.000	0.00	10.09
**ENERGY ~ BodySize * RangeSize**	7	-228.65	471.85	**0.566**	13.19	15.95
ENERGY ~ BodySize + RangeSize	6	-229.99	472.39	0.434	12.69	13.94
ENERGY ~ 1	3	-263.40	532.91	0.000	0.00	12.96
**HUMAN ~ BodySize * RangeSize**	7	-303.83	622.22	**1.000**	20.19	26.90
HUMAN ~ BodySize + RangeSize	6	-313.67	639.76	0.000	16.94	25.34
HUMAN ~ 1	3	-462.15	930.42	0.000	0.00	20.04

Supplementary Table 3 (1 of 2). Results of the generalized linear mixed-effect models predicting the total deviance explained in fish occurrence patterns by boosted regression trees (DE) and the relative contribution of biogeography-, area- and energy-related correlates and human pressure as a function of individual biological traits including: maximum adult total length (*BodySize*, in cm), extent of occurrence (*RangeSize*, in 10^6 km^2), diet, schooling behaviour, diel activity and home range (see description in Supplementary Table 5). Models included a random effect coding for the genus nested within the family (1|Family/Genus) to account for phylogenetic relationships among taxa. Shown are the number of parameters (*k*), the log-likelihood (*LogL*), Akaike's information criterion corrected for small sample sizes (AIC_c) and AIC_c weights ($wAIC_c$), the marginal R^2 (R_m) and conditional R2 (R_c). In each model set, models are ordered by decreasing $wAIC_c$, with the best model based on $wAIC_c$ indicated in bold.

Model	*k*	LogL	AIC_c	$wAIC_c$	R_m	R_c
DE ~ RangeSize	5	-818.43	1647.16	1.000	17.94	41.18
DE ~ BodySize	5	-832.41	1675.12	0.000	20.55	35.86
DE ~ 1	4	-841.65	1689.41	0.000	0.00	31.36
DE ~ Diet	9	-850.49	1733.47	0.000	0.98	30.59
DE ~ Schooling	7	-856.76	1736.87	0.000	0.59	30.28
DE ~ Activity	5	-861.19	1736.94	0.000	0.19	30.19
DE ~ HomeRange	5	-861.36	1737.27	0.000	0.38	29.62
BIOGEOGRAPHY ~ BodySize	5	-764.95	1540.20	0.872	5.66	14.05
BIOGEOGRAPHY ~ RangeSize	5	-767.53	1545.36	0.066	0.30	10.96
BIOGEOGRAPHY ~ 1	4	-769.73	1545.57	0.059	0.00	11.01
BIOGEOGRAPHY ~ Activity	5	-769.02	1552.60	0.002	0.69	12.45
BIOGEOGRAPHY ~ Diet	9	-761.87	1556.23	0.000	0.62	13.41
BIOGEOGRAPHY ~ HomeRange	5	-770.86	1556.27	0.000	0.08	11.97
BIOGEOGRAPHY ~ Schooling	7	-767.12	1557.58	0.000	0.30	10.13
AREA ~ RangeSize	5	-780.41	1571.11	1.000	12.96	15.31
AREA ~ BodySize	5	-792.23	1594.77	0.000	0.44	13.32
AREA ~ 1	4	-794.50	1595.12	0.000	0.00	10.09
AREA ~ Diet	9	-787.18	1606.85	0.000	0.97	11.58
AREA ~ HomeRange	5	-796.23	1607.02	0.000	0.63	11.23
AREA ~ Activity	5	-796.39	1607.34	0.000	0.29	10.24
AREA ~ Schooling	7	-792.15	1607.63	0.000	0.90	10.43

Supplementary Table 3 (2 of 2).

Model	*k*	LogL	AIC$_c$	*w*AIC$_c$	R$_m$	R$_c$
ENERGY ~ BodySize	5	-727.85	1461.82	0.398	0.00	13.66
ENERGY ~ RangeSize	5	-725.98	1462.26	0.320	0.00	13.03
ENERGY ~ 1	4	-726.12	1462.54	0.278	0.00	12.97
ENERGY ~ Activity	5	-728.86	1472.27	0.002	0.52	12.50
ENERGY ~ Diet	9	-720.93	1474.35	0.001	1.22	13.71
ENERGY ~ HomeRange	5	-730.58	1475.71	0.000	0.01	12.02
ENERGY ~ Schooling	7	-726.33	1476.01	0.000	0.64	11.31
HUMAN ~ RangeSize	5	-698.64	1407.58	1.000	12.65	24.12
HUMAN ~ BodySize	5	-708.66	1427.61	0.000	9.81	21.78
HUMAN ~ 1	4	-713.62	1433.37	0.000	0.00	20.04
HUMAN ~ HomeRange	5	-727.15	1468.86	0.000	1.11	20.46
HUMAN ~ Activity	5	-728.84	1472.24	0.000	0.04	20.11
HUMAN ~ Schooling	7	-724.52	1472.38	0.000	1.01	19.48
HUMAN ~ Diet	9	-721.31	1475.11	0.000	0.56	19.39

Supplementary Table 4 Results of null models, indicating no evidence for a relationship between (*i*) the variation in fish occurrence patterns (DE_{BRT}) and body or range size, or between (*ii*) the relative contribution of biogeography, energy and human pressure and body or range sizes. In each model set, models are ordered by decreasing $wAIC_c$, with the best model based on $wAIC_c$ in bold. In all cases (except for the *Area* model set) the model including an intercept only outperforms other models based on $wAIC_c$. The *area* model was the only one for which we found support for an interaction between body and range sizes, which explained only ~ 2 % deviance in the relative contribution of area correlates to the variation explained in fish occurrence patterns.

Model	*k*	LogL	AIC_c	$wAIC_c$	DE_{GLMM}
**DE_{BRT} ~ 1**	3	746.97	-1487.74	**1.000**	0.00
DE_{BRT} ~ BodySize + RangeSize	6	731.34	-1449.94	0.000	2.09
DE_{BRT} ~ BodySize * RangeSize	7	724.81	-1434.63	0.000	2.97
**BIOGEOGRAPHY ~ 1**	3	-451.28	908.77	**0.978**	0.00
BIOGEOGRAPHY ~ BodySize + RangeSize	6	-451.91	916.55	0.020	0.14
BIOGEOGRAPHY ~ BodySize * RangeSize	7	-453.08	921.15	0.002	0.40
**AREA ~ BodySize * RangeSize**	7	-450.27	915.53	**0.967**	2.00
AREA ~ BodySize + RangeSize	6	-455.07	922.88	0.025	0.95
AREA ~ 1	3	-459.45	925.10	0.008	0.00
**ENERGY ~ 1**	3	-421.46	849.12	**0.715**	0.00
ENERGY ~ BodySize + RangeSize	6	-419.16	851.06	0.272	0.55
ENERGY ~ BodySize * RangeSize	7	-421.06	857.11	0.013	0.09
**HUMAN ~ 1**	3	-414.92	836.04	**0.961**	0.00
HUMAN ~ BodySize + RangeSize	6	-414.91	842.57	0.037	0.00
HUMAN ~ BodySize * RangeSize	7	-416.47	847.94	0.003	0.38

Supplementary Table 5. Characteristics of individual reef fish datasets: total number of locations, total number of replicates (transects or stationary point counts [SPC]), survey area of each replicate, depth range, methods, sampling period and key references describing the sampling technique. When the sampling period extended beyond the publication year of the reference, the same (standardized) sampling techniques as described in the reference were used throughout.

Dataset	No locations	Total no replicates	Survey area (m^2)	Depth range (m)	Survey methods (summary)	Sampling period	References
AIMS	46	685	50-250	6-9	[Transects] 3 replicate 50-m long transects at each location (reef). Transect are 1-m wide for small site-attached species, 5-m wide for others.	2003-2007	1
CRED	59	1241	177	1-30	[SPC] 2 contiguous 15-m diameter plots based on stratified sampling in 3 depth zones for each location	2010-2011	2
GASPAR	102	4370	50-500	1-30	[Transect] 25 m or 50 m line transects (distance sampling truncated to 5 m, or 4-m wide for small site-attached species, 8-m wide for others)	1991-2011	3-11
PROCFish	197	1650	500	1-15	[Transect] 50 m line transects (distance sampling) with at least 6 transects per reef type at each site	2002-2009	12,13
RLS	390	1263	500	1-25	[Transect] 2 contiguous 5 x 50-m long transect blocks at each depth stratum, with 1 to 5 depth strata surveyed at each site.	2008-2012	14,15
WCS	106	247	500	1-20	[Transect] 1-4 transects at each site, either 5 x 100-m or 10 x 50-m long	1991-2012	16
WF	6	372	56-128	0-20	[SPC] 3 points per station (10 m radius max and 5 m radius min). One on the reef flat, one on the reef crest and one at the bottom of the reef slope.	2000	17,18

With AIMS: Australian Institute of Marine Science; CRED: Coral Reef Ecosystem Division, University of Hawaii; GASPAR: General Approach to SPecies-Abundance Relationships; PROCFish: Pacific Regional Oceanic and Coastal Fisheries development programme, Secretariat of the Pacific Community (SPC); RLS: Reef Life Survey; WCS: Wildlife Conservation Society; WF: Wallis and Futuna.

Supplementary Table 6. Correlates of reef fish occurrence related to energy, area, biogeography and human pressure used in the boosted regression trees. For factors (i.e. *THREAT* and *THREAT_STR*) the medium is given instead of the mean.

Hypothesis	Variable code	Variable description	Unit	Mean	Range	Source
**Biogeography**	Dist2Land	Distance to nearest land mass > 10^5 m^2	km	619.7	0-3880	Calculated using ArcGis 10.0
	Dist2Shelf	Distance to edge of continental shelf	km	36.4	0-977	
	Dist2Edge_km	Distance to range edge	km	1170.0	0-9996	
	Dist2Edge_rel	Relative distance to range edge ϵ [0,1]		0.1	0-1	
	X_rel	Relative longitude [-1;1] within range		0.7	0-1	
	Y_rel	Relative latitude [-1;1] within range		0.4	0-1	
	Dist2CT	Distance to Coral Triangle	km	6796.3	0-13476	
**Area**	ReefArea	Total area of the surveyed (or nearest) reef	km^2	63.4	0-363	Calculated using ArcGis 10.0 from the Reefs at Risk global reef layer[6] available at http://www.wri.org/resources/data-sets/reefs-risk-revisited
	ReefPerim	Total perimeter of the surveyed (or nearest) reef	km	78.1	0-535	
	ReefArea10	Total reef area within a 10-km radius	km^2	29.0	0-188	
	ReefArea50	Total reef area within a 50-km radius	km^2	206.5	0-1555	
	ShelfArea50	Total area of the continental shelf (sea-bottom down to 200 m depth) within a 50-km radius	km^2	1437.7	0-7854	SRTM30_PLUS bathymetry (Shuttle Radar Topography Mission) available at http://topex.ucsd.edu/WWW_html/srtm30_plus.html
**Energy**	SST_mean	Mean annual sea surface temperature (SST)	°C	26.6	21.3-31.3	MODIS Aqua monthly climatology at a 9-km resolution (11u daytime): http://oceancolor.gsfc.nasa.gov/cgi/l3
	SST_sdev	Seasonality in SST (standard deviation in monthly SST)	°C	1.5	0-10.9	
	SST_min	Minimum (winter) annual SST	°C	25.3	18.2-30	
	SST_max	Maximum	°C	29.3	23.7-	

		(summer) annual SST			32.7	
	Chla_mean	Mean annual Chlorophyll a concentration [Chl *a*]	mg m^{-3}	0.4	0-4.6	
	Chla_sdev	Seasonality in [Chl *a*] (standard deviation in monthly [Chl *a*])	mg m^{-3}	0.1	0-3.9	
	Chla_min	Minimum (winter) annual [Chl *a*]	mg m^{-3}	0.3	0-4.7	
	Chla_max	Maximum (summer) annual [Chl *a*]	mg m^{-3}	0.5	0-4.9	
**Human pressure**	THREAT	Present local threat to coral reefs[19]		medium	low-very high	http://www.wri.org/resources/data-sets/reefs-risk-revisited
	THREAT_STR	Present integrated threat to coral reefs that accounts for past thermal stress[19] (1998-2007)		high	low-very high	
	HUM_IMPACT	Human impact[20] ϵ [0,90]		12.2	0-64	http://comlmaps.org/how-to/layers-and-resources/human-impact/human-impacts-to-marine-ecosystems
	OHI	Ocean health Index[21] ϵ [0,100]		61.8	46-73	ftp://ohi.nceas.ucsb.edu/pub/data/2012/
	OHI_FIS	OHI subcomponent 'Food provision/fisheries'		24.6	0-87	
	OHI_AO	OHI subcomponent 'Artisanal fisheries'		89.6	60-97	
	OHI_LE	OHI subcomponent 'Coastal livelihoods and economies'		85.5	40-100	
	OHI_SP	OHI subcomponent 'Sense of place'		54.6	31-85	
	OHI_BD	OHI subcomponent 'Biodiversity'		80.0	73-90	
	OHI_CP	OHI subcomponent 'Coastal Protection'		71.5	32-100	

Supplementary references

[1] A. R. Halford and A. A. Thompson, *Visual census surveys of reef fish. Long term monitoring of the Great Barrier Reef Standard Operational Procedure Number 3.* (Australian Institute of Marine Science, Townsville, 1996).

[2] P. Ayotte*, et al.*, *Coral Reef Ecosystem Division standard operating procedures: data collection for rapid ecological assessment fish surveys* (Pacific Islands Fish. Cent., Natl. Mar. Fish. Serv., NOAA, Honolulu, HI USA, 2011).

[3] Kulbicki, M. *et al.* Les communautes de poissons lagonaires dans les atolls des Tuamotu, principaux resultats du programme TYPATOLL (1995-1996). *Noumea, IRD, Doc. Sci. Tech.* **113**, 125 (2000).

[4] P. Labrosse, M. Kulbicki, and J. Ferraris, *Underwater visual fish census surveys. Proper use and implementation. Reef resource assessment tools.* (Secretariat of the Pacific Community, Noumea, New Caledonia, 2002).

[5] Planes, S. *et al.* Stability of coral reef fish assemblages impacted by nuclear tests. *Ecology* **86**, 2578-2585 (2005).

[6] Mellin, C. *et al.* Diversity of coral reef fish assemblages: modelling of the species richness spectra from multi-scale environmental variables in the Tuamotu Archipelago (French Polynesia). *Ecol. Model.* **198**, 409-425 (2006).

[7] Mellin, C. *et al.* Natural and anthropogenic influences on the diversity structure of reef fish communities in the Tuamotu Archipelago (French Polynesia). *Ecol. Model.* **218**, 182-187 (2008).

[8] M. Kulbicki*, et al.*, *Caracteristiques des peuplements de poissons de recif des iles hautes de Polynesie francaise: une revue des donnees disponibles.* (Rapport de Convention EPHE-AAMP, Perpignan (France), 2009).

[9] MacNeil, M. A. *et al.* A confederacy of processes: hierarchical drivers of reef-fish metacommunity structure. *Ecology* **90**, 252-264 (2009).

10 Planes, S. *et al.* Environmental determinants of coral reef fish diversity across several French Polynesian atolls. *CR Biologies* **335**, 417-423 (2012).

11 Friedlander, A. M. *et al.* Habitat-specific characterization of the fish assemblage at a piscivore-dominated, pristine atoll in the central Pacific. *Mar. Ecol. Prog. Ser.* **410**, 219-231 (2010).

12 Pinca, S. *et al.* Relative importance of habitat and fishing in influencing reef fish communities across seventeen Pacific Islands Countries and Territories. *Fish and Fisheries* **13**, 361-379 (2012).

13 D'agata, S. *et al.* Human-mediated loss of phylogenetic and functional diversity in coral reef fishes. *Curr. Biol.* **24**, 555-560 (2014).

14 Edgar, G. J. & Stuart-Smith, R. D. Systematic global assessment of reef fish communities by the Reef Life Survey program. *Sci. Data* **1***,* 140007 (2014).

15 Stuart-Smith, R. D. *et al.* Integrating abundance and functional traits reveals new global hotspots of fish diversity. *Nature* **501**, 539-542 (2013).

16 McClanahan, T. R. Kenyan coral-reef lagoon fish - effects of fishing, substrate complexity, and sea-urchins. *Coral Reefs* **13**, 231-241 (1994).

17 Steven K. Thompson, *Sampling, 3rd edition* (John Wiley & Sons, Inc, 2012).

18 Wantiez, L. & Chauvet, C. First data on community structure and trophic networks of uvea coral reef fish assemblages (Wallis and Futuna, South Pacific Ocean). *Cybium* **27**, 83-100 (2003).

19 L. Burke*, et al.*, *Reefs at Risk Revisited* (World Resources Institute, Washington, DC, 2011).

20 Halpern, B. S. *et al.* A global map of human impact on marine ecosystems. *Science* **319**, 948-952 (2008).

21 Halpern, B. S. *et al.* An index to assess the health and benefits of the global ocean. *Nature* **488**, 615-620 (2012).
